# Werner Helicase Control of Human Papillomavirus 16 E1-E2 DNA Replication Is Regulated by SIRT1 Deacetylation

**DOI:** 10.1128/mBio.00263-19

**Published:** 2019-03-19

**Authors:** Dipon Das, Molly L. Bristol, Nathan W. Smith, Claire D. James, Xu Wang, Pietro Pichierri, Iain M. Morgan

**Affiliations:** a Department of Oral and Craniofacial Molecular Biology, VCU Philips Institute for Oral Health Research, Virginia Commonwealth University School of Dentistry, Richmond, Virginia, USA; b Department of Environment and Health, Istituto Superiore di Sanità, Rome, Italy; c VCU Massey Cancer Center, Richmond, Virginia, USA; University of Michigan-Ann Arbor

**Keywords:** DNA repair, DNA replication, E1-E2, SIRT1, WRN, cervical cancer, head and neck cancer, human papillomavirus, life cycle

## Abstract

Human papillomaviruses (HPV) are double-stranded DNA viruses causative in a host of human diseases, including several cancers. Following infection, two viral proteins, E1 and E2, activate viral replication in association with cellular factors and stimulate the DNA damage response (DDR) during the replication process. E1-E2 uses homologous recombination (HR) to facilitate DNA replication, but an understanding of host factors involved in this process remains incomplete. Previously, we demonstrated that the class III deacetylase SIRT1, which can regulate HR, is recruited to E1-E2-replicating DNA and regulates the level of replication. Here, we demonstrate that SIRT1 promotes the fidelity of E1-E2 replication and that the absence of SIRT1 results in reduced recruitment of the DNA repair protein Werner helicase (WRN) to E1-E2-replicating DNA. CRISPR/Cas9 editing demonstrates that WRN, like SIRT1, regulates the quantity and fidelity of E1-E2 replication. This is the first report of WRN regulation of E1-E2 DNA replication, or a role for WRN in the HPV life cycle. In the absence of SIRT1 there is an increased acetylation and stability of WRN, but a reduced ability to interact with E1-E2-replicating DNA. We present a model in which E1-E2 replication turns on the DDR, stimulating SIRT1 deacetylation of WRN. This deacetylation promotes WRN interaction with E1-E2-replicating DNA to control the quantity and fidelity of replication. As well as offering a crucial insight into HPV replication control, this system offers a unique model for investigating the link between SIRT1 and WRN in controlling replication in mammalian cells.

## Abstract (precis)

HPV16 is the major viral human carcinogen responsible for between 3 and 4% of all cancers worldwide. Following infection, this virus activates the DNA damage response (DDR) to promote its life cycle and recruits DDR proteins to its replicating DNA in order to facilitate homologous recombination during replication. This promotes the production of viable viral progeny. Our understanding of how HPV16 replication interacts with the DDR remains incomplete. Here, we demonstrate that the cellular deacetylase SIRT1, which is a part of the E1-E2 replication complex, regulates recruitment of the DNA repair protein WRN to the replicating DNA. We demonstrate that WRN regulates the level and fidelity of E1-E2 replication. Overall, the results suggest a mechanism by which SIRT1 deacetylation of WRN promotes its interaction with E1-E2-replicating DNA to control the levels and fidelity of that replication.

## INTRODUCTION

Human papillomaviruses (HPV) are causative agents in human diseases ranging from genital warts to ano-genital and oropharyngeal cancers (1). HPV16 is causative in around 50% of cervical cancers and 90% of HPV-positive oropharyngeal (HPV+OPC) cancers (1, 2). HPV are thought to infect stem cells in the basal layer of the epithelium (3), and following infection, the ∼8-kbp circular viral DNA is delivered to the nucleus, where cellular factors activate viral transcription (4). This results in expression of the viral genes, including the E6 and E7 oncogenes. E7 binds to Rb and other pocket proteins and disrupts the control of E2F transcription factors, while E6 binds to and mediates the degradation of p53 (5); the overall result is to promote proliferation of the infected cell. This ultimately results in differentiation that is required for viral production in the upper layers of the differentiated epithelium (3, 6). In cancer, the infected cell fails to fully differentiate and continues to proliferate, resulting in the accumulation of genetic damage promoting cell transformation and progression to tumorigenesis.

HPV encodes two proteins, E1 and E2, that are required to replicate the viral genome in conjunction with host factors (7–13). The E2 protein forms homodimers and binds to 12-bp palindromic sequences surrounding the A/T-rich origin of replication (14). Via a protein-protein interaction in the amino-terminal domain of E2, the E1 helicase is recruited to the viral genome; E1 then forms a di-hexameric helicase and interacts with cellular polymerases to initiate replication of the viral genome (15). Following infection, the virus establishes itself at around 20 to 50 copies per cell. The infected cells then proliferate through the epithelium, where there is a maintenance phase of DNA replication that keeps the viral genome copy number at 20 to 50. In the differentiated layer of the epithelium, there is an amplification phase of viral replication during which the viral genome copy number increases to around 1,000. The L1 and L2 structural proteins are then expressed, and viral particles that egress from the upper layers of the epithelium are formed (3). A full understanding of the host proteins that regulate viral replication at all stages of the viral life cycle remains to be elucidated.

We identified the DNA damage repair and replication protein TopBP1 as a cellular partner protein for HPV16 E2 and demonstrated that this interaction is involved in E1-E2 replication and the viral life cycle (11–13, 16). TopBP1 is an essential gene (17) due to its role in a host of nucleic acid metabolism processes that include DNA damage recognition, signaling, and repair (18–22) as well as DNA replication initiation (16, 23–33) and regulation of transcription (34–37). To expand our understanding of cellular proteins regulating E1-E2 DNA replication, we investigated the role of known TopBP1 interactors in this process. The class III deacetylase SIRT1 regulates TopBP1 function following replication and metabolic stress via regulation of TopBP1 acetylation status (38, 39). SIRT1 can also regulate the acetylation status of other proteins involved in DNA replication initiation (40); therefore, we postulated that SIRT1 may be able to regulate E1-E2 DNA replication. We demonstrated that SIRT1 interacts with both E1 and E2 and is recruited to E1-E2-replicating DNA and that CRISPR/Cas9 editing of SIRT1 resulted in elevated E1-E2 replication, perhaps via increased acetylation of E2 (41). SIRT1 has been shown to play a similar role in mammalian DNA replication (42); phosphorylation of SIRT1 on threonine 530 promotes SIRT1 association with replication origins, facilitates replication fork elongation, and is required to maintain genome integrity following replication stress.

E1-E2 DNA replication activates the DNA damage response (DDR) (43–48) and recruits a variety of cellular factors required for homologous recombination (HR) to the viral genome (11, 12, 47, 49, 50); it has been proposed that E1-E2 DNA replication proceeds via HR in the presence of an active DDR (51). The reason that the virus activates the DDR is related to the mode of E1-E2 replication, where initiation is not restricted to only once per cell cycle; reinitiation of genomes already undergoing replication results in torsional stress and potentially clashes of DNA replication forks that would activate the DDR (52). Exploiting HR to maintain the fidelity of E1-E2 replication would therefore promote the generation of successful viral progeny. The E1-E2-interacting factor SIRT1 (41) plays a role in HR. NBS1, a member of the MRN (Mre11-Rad50-Nbs1) complex, is a SIRT1 substrate, and deacetylation of NBS1 by SIRT1 is required for ATM phosphorylation of NBS1, promoting the formation of the MRN complex (53). This MRN complex is required for initiating DNA resection at damaged DNA sites in order to promote HR (25, 54). Recruitment of MRN components is required for efficient HPV DNA replication (47, 49, 50). SIRT1 increases global HR function (55), although precisely how SIRT1 does this is not known. SIRT1 is required for the successful amplification of HPV31 during epithelial cell differentiation and can complex with the viral genome during this process (56). Another DDR protein regulated by SIRT1 is the Werner helicase (WRN). Deacetylation by SIRT1 regulates WRN stability and promotes its role in HR (57–60). The WRN gene is unique in encoding both a 3′-to-5′ exonuclease and 3′-to-5′ helicase activity and has a role in promoting genomic stability; notably, Werner syndrome patients have an increased frequency of cancer incidence (61–64). WRN, like SIRT1 (55), is also involved in regulating telomere ends during replication (65), further linking these two proteins.

Here, we report that E1-E2 DNA replication in the absence of SIRT1 has an increased mutation frequency compared with that of wild-type SIRT1 cells. In the absence of SIRT1, there is an enhanced acetylation of WRN, and this acetylated WRN has a reduced recruitment to the E1-E2-replicating DNA. CRISPR/Cas9 removal of WRN results in elevated levels of E1-E2 replication and an increased mutation frequency, a phenotype identical to that observed in the absence of SIRT1. Overall, these results suggest that E1-E2 replication stimulates a DDR activating the deacetylation enzyme function of SIRT1, which then deacetylates WRN and promotes the interaction of this repair protein with E1-E2-replicating DNA. The recruitment of WRN controls both the levels and the fidelity of E1-E2 DNA replication. We propose that this SIRT1-WRN interaction also plays an important role in host DNA replication and that E1-E2 replication can serve as a model to dissect this process in mammalian cells.

## RESULTS

### SIRT1 controls the fidelity of E1-E2 DNA replication and the recruitment of WRN to the replicating DNA.

**FIG 1:**
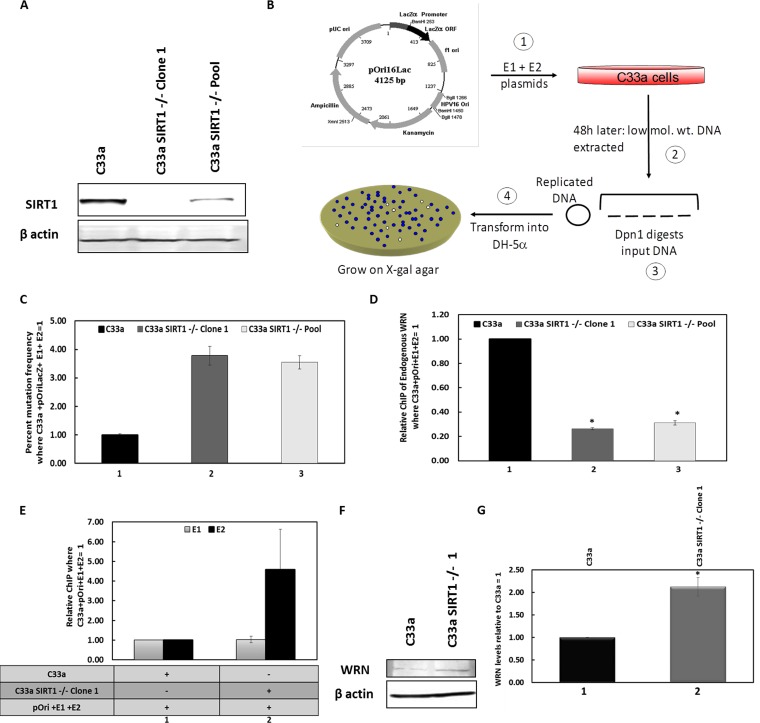
Absence of SIRT1 results in mutagenic E1-E2 DNA replication and a reduction in recruitment of WRN to the replicating DNA. (A) C33a cells with CRISPR/CAS9 editing of SIRT1 expression demonstrate a reduction in SIRT1 expression in a clonal line (Clone 1) and in pooled cells (Pool). (B) Graphic description of our E1-E2 mutagenesis assay, a key component of this report. ORF, open reading frame; ori, origin of replication. In step 1, E1 and E2 expression plasmids are cotransfected with pOri16Lac, which contains the HPV16 origin of replication and the *lacZ* gene. In step 2, 48 h later, low-molecular-weight (low mol. wt.) DNA is harvested from the cells and digested with DpnI (3), which digests the transfected DNA but not the replicated DNA. The replicated DNA is then transfected into DH10B or DH5α cells (4) and plated on agar with X-gal and kanamycin. White colonies indicate replicated molecules that have picked up mutations in the *lacZ* gene (66). (C) E1-E2 DNA replication in the absence of SIRT has a significantly enhanced mutagenic phenotype. In both C33a SIRT1^–/–^ clone 1 and pool cells, there is an enhanced mutagenesis compared with that of wild-type cells (compare lanes 2 and 3 with lane 1). This increase is statistically significant (*P* value is less than 0.05), and standard error bars are shown. In the absence of E1 or E2, there are no bacterial colonies detected, as there is no replication. The histogram depicts the results of three independent experiments. (D) In the absence of SIRT1, there is a reduction in recruitment of WRN to the E1-E2-replicating DNA. Chromatin was prepared from the cells, and ChIP assays were carried out with a WRN antibody; the results are expressed relative to the levels in wild-type C33a cells, which equal 1. The results presented are a summary of results from three independent experiments. Figure S1B and C describe the controls for these experiments. The reduction in WRN recruitment is statistically significant, as indicated with an asterisk (the *P* value was less than 0.05), and standard error bars are shown. (E) There is no significant difference in E1 and E2 recruitment to the replicating DNA in the absence of SIRT1, although E2 levels do trend higher, presumably due to the elevated levels of E2 in the absence of SIRT1 (41). Shown is a summary of the results from at least 3 independent experiments, and standard error bars are presented. (F) In the absence of SIRT1, there is an increased level of endogenous WRN. (G) This experiment was repeated, and the results were quantitated; there is a significant increase (*) of WRN in the absence of SIRT1 (*P* value was less than 0.05; standard error bars are shown).

Previously, we demonstrated that SIRT1 is a member of the HPV16 E1-E2 DNA replication complex, deacetylates and destabilizes E2, and controls the levels of replication (41). To do this, we used CRISPR/Cas9-edited cells (Fig. 1A). Given the role of SIRT1 in regulating the DDR and HR, we investigated whether SIRT1 was involved in regulating the fidelity of E1-E2 replication. To monitor E1-E2 replication fidelity, we employed our assay in which E1 and E2 replicate an HPV16 origin-containing plasmid that includes the *lacZ* gene. A description of this assay is given in Materials and Methods and in Fig. 1B; we have previously used this assay to investigate E1-E2 DNA replication (48, 66). It has demonstrated that HPV16 E1-E2 DNA replication uses translesion synthesis to bypass replication polymerases on UV-damaged DNA (66) and that replication in the presence of DNA-damaging agents is mutagenic (48). We now demonstrate that deletion of SIRT1 from C33a cells resulted in an elevation in mutation frequency of 3- to 4-fold (Fig. 1C). Restoration of SIRT1 expression during E1-E2 DNA replication in the SIRT1 CRISPR knockout cells resulted in a restoration of E1-E2 DNA replication fidelity (see Fig. S1A in the supplemental material). This restoration, combined with SIRT1 overexpression restoring wild-type E1-E2 replication levels in the SIRT1 CRISPR/Cas9 knockout cells (41), demonstrates that the replication effects following SIRT1 depletion are not due to off-target effects of the CRISPR/Cas9 targeting sequences.

10.1128/mBio.00263-19.1 FIG S1 (A) This figure demonstrates that overexpression of FLAG-SIRT1 in C33a SIRT1^–/–^ clone 1 cells restores fidelity to E1-E2 DNA replication. Experiments in duplicate were carried out as described for Fig. 1C. Overexpression of FLAG-SIRT1 in C33a wild-type cells had no effect on the fidelity of replication; therefore, this reduction in mutation frequency by FLAG-SIRT1 is observed only in cells that have no SIRT1 expression. There is a significant increase in mutations in the absence of SIRT1 (*) and a corresponding significant decrease (^) following FLAG-SIRT1 expression (*P* values were less than 0.05; standard error bars are shown). (B) This is a control for Fig. 1D, which describes WRN ChIP of E1-E2-replicating DNA. This figure demonstrates that the signal obtained in the ChIP experiments when no E1 or E2 is expressed is negligible. There is a significant increase in signal (*) in the presence of E1-E2 (*P* value was less than 0.05; standard error bars are present but too small to see). Expression of either replication protein by itself also does not enhance the signal (11, 41). The experiment was carried out in triplicate. (C) This panel represents a control for Fig. 1D, which describes WRN ChIP of E1-E2-replicating DNA. This figure demonstrates the signal obtained with a control antibody (rabbit serum); there is no increase in signal with the presence of E1 and E2, and this represents a background signal (standard error bars are shown). This demonstrates the specificity of the WRN signal in Fig. 1D. Experiments represented in panels B and C were carried out in triplicate. (D) This panel represents a control for Fig. 1E, which describes E1-E2 levels on replicating DNA in C33a wild-type and SIRT1^–/–^ clone 1 cells. The results presented are for the E1 and E2 proteins, respectively, in C33a wild-type cells (a hemagglutinin [HA] antibody is used to detect the HA-tagged E1 protein) and demonstrate that in the absence of E1 and E2, there is a dramatic reduction in the signal obtained (standard error bars are shown). There is a significant increase in signal in the presence of E1and E2 (*; *P* value is less than 0.05). The experiment was carried out in triplicate. (E) This panel represents a partner figure for Fig. 1F and G and shows the WRN RNA levels in C33a wild-type and SIRT1^–/–^ clone 1 and pool cells. The results show that WRN RNA levels are consistent in all cells, demonstrating that the reduction in protein level (Fig. 1G) is posttranscriptional (standard error bars are shown, and this represents the summary of results of three independent experiments). Download FIG S1, TIF file, 6.2 MB. Copyright © 2019 Das et al. 2019 Das et al. This content is distributed under the terms of the Creative Commons Attribution 4.0 International license.

We then investigated whether this reduced replication fidelity in the absence of SIRT1 was due to a failure to recruit any HR protein to the E1-E2-replicating DNA. SIRT1 can regulate the recruitment of NBS1 to damaged DNA via deacetylation (53, 67), and the MRN complex is required for E1-E2 DNA replication (47, 49, 50), but we did not see any reduction in NBS1 recruitment to E1-E2-replicating DNA in the absence of SIRT1 (not shown). However, chromatin immunoprecipitation (ChIP) analysis determined that there was a significant reduction in the recruitment of the SIRT1 substrate Werner helicase (WRN) to E1-E2-replicating DNA in the absence of SIRT1 (Fig. 1D). The results are presented as the signal obtained in the presence of E1 and E2 expression along with an HPV16 origin plasmid (pOri) in wild-type C33a cells, which equals 1. The controls for this experiment are presented in Fig. S1B and S1C; Fig. S1B demonstrates dramatically enhanced WRN recruitment to pOri only in the presence of the E1-E2 replication proteins, while Fig. S1C demonstrates no increased signal to pOri with control antibodies irrespective of E1-E2 expression and cell line. To confirm that this difference was not due to transfection efficiency, we carried out ChIP assays for E1 and E2 in C33a wild-type and C33a SIRT1^–/–^ clone 1 cells (Fig. 1E). The control for this figure is provided in Fig. S1D. There is no difference in the levels of E1 binding to the replicating DNA between the two cell types, while there is an increase in the E2 levels, perhaps due to the elevated levels of E2 in the absence of SIRT1 (41). This is the first report of WRN being involved in E1-E2 DNA replication; WRN is a known substrate of SIRT1 (57, 60, 68) and a DDR protein (61, 62, 64, 68). The levels of WRN in the absence of SIRT1 are elevated in C33a cells (Fig. 1F and G); therefore, the reduction of WRN recruitment to E1-E2-replicating DNA in the absence of SIRT1 is not due to a reduction in the overall levels of the WRN protein present in C33a cells in the absence of SIRT1. Moreover, this was not due to a decrease in WRN RNA levels (Fig. S1E); therefore, SIRT1 regulates the expression of WRN posttranscriptionally.

### WRN regulates the levels and fidelity of E1-E2 DNA replication.

**FIG 2:**
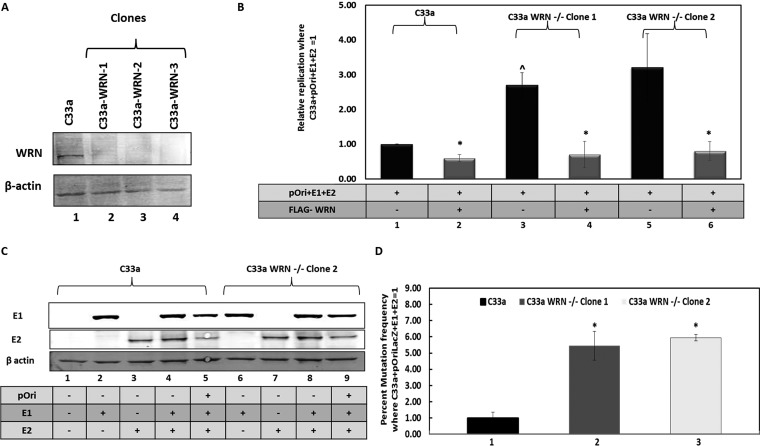
Deletion of WRN generates a phenotype similar to that after the loss of SIRT1. (A) CRISPR/Cas9 was used to generate clonal cell lines (lanes 2 to 4) that lacked WRN expression. (B) E1-E2 replication levels were elevated in the absence of WRN (compare lanes 3 and 5 with lane 1), while overexpression of wild-type WRN repressed replication (lanes 2, 4, and 6). In the WRN CRISPR cells (lanes 3 to 6), FLAG-WRN overexpression resulted in replication levels similar to those in C33a wild-type cells with FLAG-WRN overexpression (compare lanes 4 and 6 with lane 2). In each cell line, FLAG-WRN resulted in a statistically significant decrease in replication (*; *P* value was less than 0.05; standard error bars are shown). There is a statistically significant increase in replication levels (^) in C33a WRN^–/–^ clone 1 cells compared with those in C33a wild-type cells (*P* value was less than 0.05). The histogram depicts the average of results from five independent experiments. (C) The expression levels of E1 and E2 are not affected by the absence of WRN. (D) The absence of WRN results in a significantly enhanced (*) mutation frequency for E1-E2 replication (compare lanes 2 and 3 with lane 1) (*P* value was less than 0.05; standard error bars are shown). The experiment was repeated three times.

To determine whether WRN could directly regulate E1-E2 replication, C33a WRN knockout cell lines were generated using CRISPR/Cas9 targeting (Fig. 2A). The clones were sequenced to confirm disruption of the WRN locus (Fig. S2A). E1-E2 DNA replication assays were carried out with C33a, C33a-WRN-1, and C33a-WRN-2 cells, and the results are expressed relative to the levels in C33a wild-type cells, equaling 1 (Fig. 2B). The control for this replication assay (Fig. S2B) demonstrates a large increase in signal when the E1-E2 proteins are expressed in C33a wild-type cells versus signal levels in control samples that do not express the viral proteins together. In these assays, neither E1 nor E2 can stimulate replication by themselves (69). WRN overexpression from a FLAG-tagged expression plasmid significantly repressed replication in C33a cells (compare lane 2 with lane 1 in Fig. 2B), while knockout of WRN resulted in a significant increase in E1-E2 DNA replication in both C33a WRN CRISPR clones (compare lanes 3 and 5 with lane 1). Coexpression of FLAG-WRN in the CRISPR knockout cells represses E1-E2 replication (compare lane 4 with lane 3 and lane 6 with lane 5) to levels observed in C33a wild-type cells (compare lanes 4 and 6 with lane 2). The FLAG-WRN is expressed equivalently between the wild-type C33a cells and the two WRN CRISPR clones (Fig. S2C). The levels of the E1-E2 proteins are not altered by the absence of WRN (Fig. 2C). The result with one of the WRN CRISPR knockout clones is shown, but similar results were confirmed with an additional clone (data not shown). These results demonstrate that WRN can regulate the levels of E1-E2 DNA replication and that this is not due to an alteration in the levels of the viral replication factors.

10.1128/mBio.00263-19.2 FIG S2 (A) The two C33a WRN CRISPR/Cas9 clones used in the experiments throughout the paper were sequenced to confirm disruption of the WRN gene at the predicted position. A fragment of WRN exon 6 is represented, and the line above the sequence demonstrates where the guide RNA targeted; both clones have mutations within this region, as predicted. (B) This panel represents a control for Fig. 2B, which measured E1-E2 replication levels in the presence and absence of WRN. The results shown are with wild-type C33a cells with and without the E1 and E2 proteins and demonstrate a significant increase (*) in signal when the replication proteins are there (*P* value is less than 0.05; standard error bars are shown). This was then used as 1 to standardize the experimental results shown in Fig. 2B. The histogram depicts the results of five independent experiments. (C) FLAG-WRN is expressed equally well in the wild-type and WRN CRISPR knockout C33a cells. (D) The experiment whose results are shown demonstrates that overexpression of FLAG-WRN in C33a WRN^–/–^ clone 1 cells restores fidelity to E1-E2 DNA replication. The experiments were carried out in duplicate as described in the legend of Fig. 2D. Overexpression of FLAG-WRN in C33a wild-type cells had no effect on the fidelity of replication; therefore, this reduction in mutation frequency by FLAG-WRN is observed only in cells that have no WRN expression. There is a significant increase in the number of mutations in the absence of WRN (*) and a corresponding significant decrease (^) following FLAG-WRN expression (*P* values were less than 0.05; standard error bars are shown). Download FIG S2, TIF file, 6.4 MB. Copyright © 2019 Das et al. 2019 Das et al. This content is distributed under the terms of the Creative Commons Attribution 4.0 International license.

WRN is a DNA repair protein involved in several aspects of preserving stalled and damaged DNA replication forks, promoting their repair and high-fidelity DNA replication (61–64). We again used our mutagenesis assay (Fig. 1B) to investigate whether the absence of WRN promoted mutagenic E1-E2 DNA replication (Fig. 2D). In two independent C33a CRISPR WRN clones, there was a significant increase in the mutation frequency detected (compare lanes 2 and 3 with lane 1). Restoration of WRN expression during E1-E2 DNA replication in the WRN CRISPR knockout cells largely restored E1-E2 DNA replication fidelity (Fig. S2D).

These results demonstrate that WRN can regulate both the levels and the fidelity of E1-E2 DNA replication. The restoration of WRN activity via cotransfected plasmids restored the replication levels to those in wild-type C33a cells and rescued the fidelity of replication in these cells. This restoration of function by addition of the wild-type WRN to the CRISPR knockout cells demonstrates that the effects of WRN knockout on replication in the CRISPR/Cas9-targeted cells are not due to off-target effects.

The results in Fig. 2 demonstrate that a WRN knockout has a phenotype similar to that of a SIRT1 knockout; both present the phenotype of an elevation of E1-E2 DNA replication that is mutagenic in nature. Moreover, Fig. 1 demonstrates that there is a lack of recruitment of WRN to E1-E2-replicating DNA in the absence of SIRT1 even though there are elevated levels of WRN with this absence of SIRT1. Since SIRT1 regulates WRN acetylation and stability, we next investigated whether control of WRN acetylation by SIRT1 is the mechanism used by SIRT1 to control WRN recruitment to the E1-E2-replicating DNA in C33a cells.

### SIRT1 controls the acetylation status and stability of WRN during E1-E2 DNA replication.

**FIG 3:**
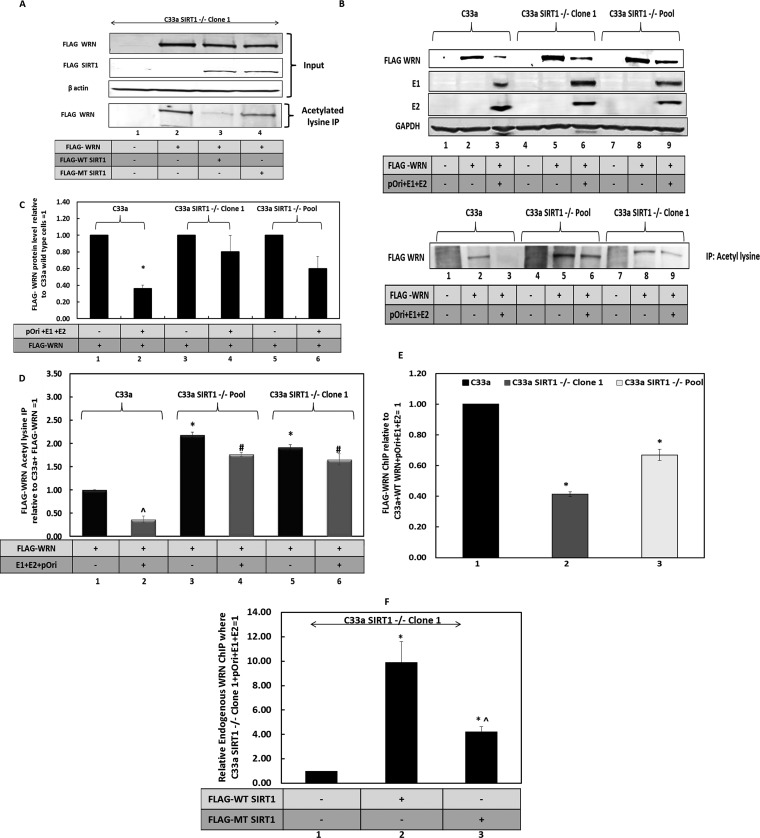
SIRT1 regulates the acetylation status of WRN. (A) FLAG-WRN, FLAG-WT SIRT1 (wild type), and FLAG-MT SIRT1 (deacetylase mutant) were coexpressed in C33a cells. The upper blots show the input levels of the proteins. The lower blot demonstrates the levels of FLAG-WRN immunoprecipitated by an acetyl lysine residue antibody. FLAG-WT SIRT1 reduces the levels of FLAG-WRN acetylation (compare lane 3 with lane 2), while FLAG-MT SIRT1 is compromised in this property (compare lane 4 with lane 3). (B) FLAG-WRN levels are reduced by the E1-E2 replication complex. In wild-type C33a cells, FLAG-WRN levels are reduced by the E1-E2 replication complex (compare lane 3 with lane 2). However, in C33a SIRT1^–/–^ clone 1 and pool cells, there is not as pronounced a reduction in FLAG-WRN levels (compare lanes 6 and 9 with lane 3). These extracts were immunoprecipitated with an acetyl lysine antibody and subjected to Western blotting for FLAG-WRN (lower panel). GAPDH, glyceraldehyde-3-phosphate dehydrogenase. (C) Quantitations of FLAG-WRN for the input blot in panel B are represented graphically and represent the summary of results from three independent experiments. There is a significant reduction in FLAG-WRN levels in wild-type C33a cells in the presence of the E1-E2 replication complex (*P* value was less than 0.05), and this reduction is largely lost in the absence of SIRT1 (compare lanes 4 and 6 with lane 2; standard error bars are shown). The results are expressed relative to levels in the absence of E1-E2, which equals 1 in each of the cell types. (D) The results obtained from the acetyl lysine IP experiments shown in the lower section of panel B were quantitated. ^ indicates a significant reduction in acetylated FLAG-WRN in the presence of the E1-E2 replication complex in C33a wild-type cells. * indicates an increase in FLAG-WRN acetylation in the absence of replication or a reduction of SIRT1 relative to the levels observed in wild-type C33a cells. # indicates an increased acetylation of FLAG-WRN in the presence of the E1-E2 replication complex but in the absence of SIRT1, compared with that of wild-type C33a cells. The significance is determined by a *P* value of less than 0.05, and standard error bars are shown. The results represent a summary of two independent experiments. (E) Even though there are elevated FLAG-WRN levels in the absence of SIRT1, there is a failure of recruitment of this protein to E1-E2-replicating DNA, as demonstrated by ChIP. The results are presented relative to those of the FLAG-WRN ChIP for wild-type C33a cells (lane 1), and there is a clear reduction in the presence of this protein on E1-E2-replicating DNA in the absence of SIRT1 (lanes 2 and 3). This difference is significant (*; *P* value was less than 0.05; standard error bars are shown). Results represent a summary of at least 3 independent experiments.

The ability of SIRT1 to deacetylate WRN was investigated in C33a cells (Fig. 3A). C33a SIRT1^–/–^ clone 1 cells (41) were transfected with FLAG-SIRT1 expression vectors encoding wild-type SIRT1 (lane 3) and a deacetylase mutant (MT) (lane 4) along with a FLAG-WRN expression vector (lanes 2–4). All of the transfected proteins were expressed (Fig. 3A, top blot). Acetylated lysine immunoprecipitation was then carried out on the cell extracts, and the resultant precipitation was blotted for the presence of FLAG-WRN. In the absence of SIRT1, FLAG-WRN is substantially acetylated (Fig. 3A, bottom blot, lane 2), while coexpression of wild-type FLAG-SIRT1 substantially reduced this acetylation (compare lane 3 with lane 2 in the bottom blot of Fig. 3A). Expression of the deacetylase mutant of SIRT1 still reduced the levels of FLAG-WRN acetylation (Fig. 3A, compare lane 4 with lane 2 in the bottom blot), although not to the same extent as wild-type SIRT1 (compare lane 4 with lane 3 in the bottom blot). These results confirm that WRN is a SIRT1 substrate in C33a cells. They also suggest that the SIRT1 H363Y mutant retains some deacetylation activity. The experiment shown in Fig. 3A was repeated, and the acetylation status of the proteins was quantitated (Fig. S3A).

10.1128/mBio.00263-19.3 FIG S3 (A) The results shown here are quantitations of the results of three independent experiments represented in Fig. 3A. The asterisk demonstrates a significant decrease in acetylation from lane 1 (*P* value was less than 0.05; standard error bars are shown). (B) This is a control for Fig. 3E, which describes FLAG-WRN ChIP of E1-E2-replicating DNA. This figure demonstrates that the signal obtained in the ChIP experiments when no E1 or E2 was expressed is negligible. Expression of either protein by itself does not enhance the signal (1, 2). The difference is significant (*; *P* value was less than 0.05; standard error bars are shown). (C) This is a control for Fig. 3E, which describes FLAG-WRN ChIP of E1-E2-replicating DNA. This panel demonstrates the signal obtained with a control antibody (rabbit serum); there is no significant increase in signal with the presence of E1 and E2, and this represents a background signal (standard error bars are shown). The results demonstrate the specificity of the FLAG-WRN signal in Fig. 3E. The results in panels B and C depict the summary of the results of three independent experiments. (D) This is a control for Fig. 3F, which describes endogenous WRN ChIP of E1-E2-replicating DNA. This figure demonstrates the signal obtained with a control antibody (rabbit serum). There is no significant increase in signal with the presence of E1 and E2, and this represents a background signal (standard error bars are shown). This demonstrates the specificity of the WRN signal in Fig. 3F. (E) This is a control for Fig. 3F, which describes endogenous WRN ChIP of E1-E2-replicating DNA. This figure demonstrates that the signal obtained in the ChIP experiments when no E1 or E2 is expressed is negligible. Expression of either protein by itself does not enhance the signal (1, 2). The difference is significant (*; *P* value was less than 0.05; standard error bars are shown). The results in panels D and E depict the summary of results of three independent experiments. (F) C33a and C33a SIRT1^–/–^ clone 1 cells were transfected with a FLAG-WRN expression vector, and the fusion protein was detected using a FLAG antibody. The images were taken using a Leica confocal microscope. The bottom panel shows a secondary antibody staining only C33a cells transfected with the FLAG-WRN expression plasmid. Download FIG S3, TIF file, 14.5 MB. Copyright © 2019 Das et al. 2019 Das et al. This content is distributed under the terms of the Creative Commons Attribution 4.0 International license.

The absence of SIRT1 increases the levels of endogenous WRN in C33a cells (Fig. 1F and G). We next wanted to determine if this was also the case when E1 and E2 are expressed. This was investigated by cotransfecting a FLAG-WRN expression vector with the viral replication factors and measuring expression levels using Western blotting (Fig. 3B, upper blot). Strikingly, the presence of the E1-E2 DNA replication complex reduced the levels of the WRN protein (compare the FLAG-WRN levels in lane 2 of Fig. 3B with those in lane 3); however, this reduction in WRN is not as pronounced in the absence of SIRT1 (compare the level of FLAG-WRN in lanes 6 and 9 with that in lane 3). The quantification is shown in Fig. 3C. The levels of WRN acetylation were also determined (bottom blot of Fig. 3B). In wild-type C33a cells, there is no acetylated FLAG-WRN in the presence of the E1-E2 replication complex (compare lane 3 with lane 2 of Fig. 3B). Since SIRT1 is recruited to the E1-E2 replication complex, this proximity may enable more-efficient deacetylation of FLAG-WRN (41). In the absence of SIRT1, there is a detectable level of acetylated WRN in the presence of the E1-E2 replication complex (Fig. 3B, lanes 6 and 9). The levels of the acetylated FLAG-WRN in the absence of SIRT1 are reflective of the overall levels of FLAG-WRN in these cells, regardless of the presence of the E1-E2 replication complex (compare the acetylated lysine immunoprecipitation [IP] bands with the levels of FLAG-WRN in Fig. 3B). Quantification of the acetylated lysine IP blots demonstrates a significant difference between the acetylation status of FLAG-WRN in the presence and that in the absence of SIRT1 (Fig. 3D). Previous studies have demonstrated that acetylated WRN has a reduced ability to bind DNA, perhaps due to the increased negative charge due to the acetyl groups. Therefore, we predicted that in the absence of SIRT1, the increased acetylation of WRN would prevent the interaction of the WRN protein with E1-E2-replicating DNA. We tested this using ChIP assays for FLAG-WRN and demonstrate that this is indeed the case; in the absence of SIRT1, there is a reduced recruitment of FLAG-WRN to the E1-E2-replicating DNA (Fig. 3E, compare lanes 2 and 3 with lane 1). Results are presented relative to the signal obtained in C33a wild-type cells, equaling 1. Controls for the ChIP experiments are shown and described in Fig. S3B and S3C.

We also wanted to determine whether the deacetylase activity of SIRT1 controlled the recruitment of WRN to the E1-E2-replicating DNA. We transfected C33a SIRT1^–/–^ clone 1 cells with the replication complex along with either the wild-type SIRT1 expression plasmid or the deacetylase MT SIRT1 expression plasmid and carried out ChIP for endogenous WRN (Fig. 3F). It is clear that wild-type SIRT1 dramatically increases the recruitment of endogenous WRN to the E1-E2-replicating DNA (Fig. 3F, compare lane 1 with lane 2), supporting the model that SIRT1 deacetylation of WRN promotes interaction with replicating DNA. MT SIRT1 was reduced in its ability to promote WRN recruitment compared with that of wild-type SIRT1 (Fig. 3F, compare lanes 2 and 3), but it still provided an increased WRN recruitment over no SIRT1 (compare lane 3 with lane 1). MT SIRT1 may have residual deacetylase activity or it may be that SIRT1 plays a structural role in recruiting WRN to replicating DNA. Figure S3D and S3E in the supplemental material provide the controls for the ChIP shown in Fig. 3F.

Previous studies have demonstrated that SIRT1 can regulate the acetylation status and nucleolar localization of WRN in response to DNA damage, and we investigated this in C33a cells and C33a SIRT1^–/–^ clone 1 (Fig. S3F) (58). In the absence of SIRT1, there is a retention of WRN to nuclear compartments that are likely nucleoli. Therefore, the failure to interact with the E1-E2-replicating DNA in the absence of WRN might be related to the negative charge that accompanies increased acetylation but also to the sequestration of WRN to the nuclear compartment, where E1-E2 replication does not occur. Both mechanisms likely contribute to the failure of WRN interaction with E1-E2-replicating DNA, and both are regulated by SIRT1 regulation of WRN acetylation status.

**FIG 4:**
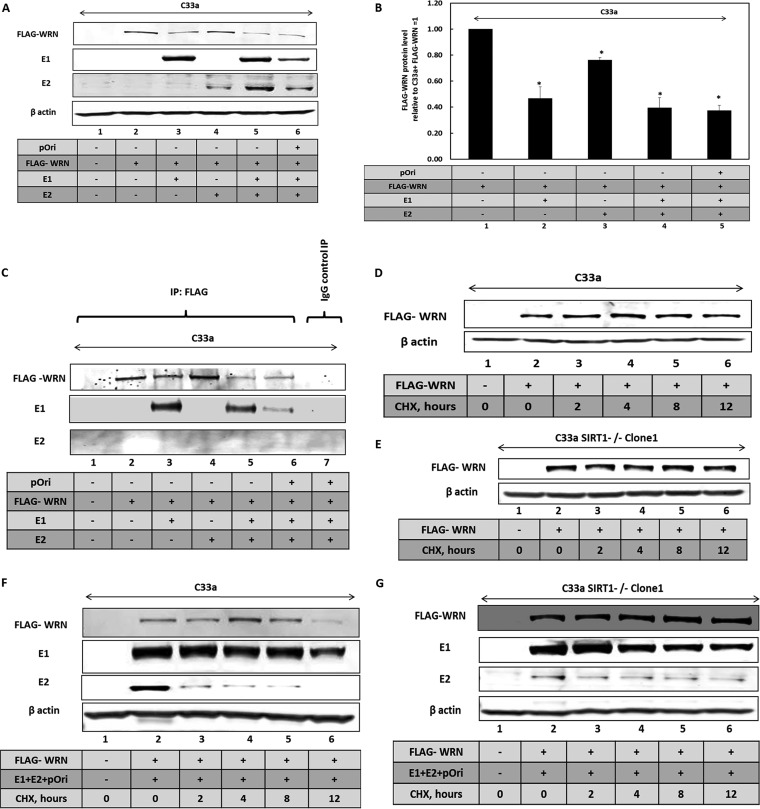
SIRT1 and the E1-E2 replication complex regulate WRN levels. (A) Western blot investigating the regulation of FLAG-WRN levels by E1 and E2. (B) The experiment represented in panel A was repeated, and the results were quantitated. The replication factors significantly downregulate the FLAG-WRN levels, as indicated by an asterisk (*P* value was less than 0.05; standard error bars are shown). The results of this quantitation for the blot in panel A are shown graphically and represent the summary of results from three independent experiments. (C) The extracts shown in panel A were immunoprecipitated by FLAG and subjected to Western blotting for the indicated proteins. FLAG-WRN interacts with E1 (lanes 3, 5, and 6) but not with E2 (lanes 4, 5, and 6). Lane 7 shows a control immunoprecipitation carried out with rabbit serum, and no immunoprecipitation of the viral factors is observed. (D) FLAG-WRN was transfected into wild-type C33a cells, and cycloheximide was added for the indicated time periods prior to cell harvesting and Western blotting of protein extracts. (E) FLAG-WRN was transfected into C33a SIRT1^–/–^ clone 1 cells and cycloheximide added for the indicated time periods prior to cell harvesting and Western blotting on protein extracts. (F) FLAG-WRN was transfected along with pOri (a plasmid containing the HPV16 origin of replication) and E1 and E2 expression plasmids into wild-type C33a cells and cycloheximide added for the indicated time periods prior to cell harvesting and Western blotting on protein extracts. (G) FLAG-WRN was transfected along with pOri (a plasmid containing the HPV16 origin of replication) and E1 and E2 expression plasmids into C33a SIRT1^–/–^ clone 1 cells, and cycloheximide was added for the indicated time periods prior to cell harvesting and Western blotting on protein extracts.

HPV16 E1 protein activates the DDR (48, 70–74), and we propose that this activates the deacetylase activity of SIRT1. This activated SIRT1 would then deacetylate WRN to promote its binding to E1-E2-replicating DNA. It is noticeable that in the presence of the E1-E2 replication proteins, there is a reduction in the levels of FLAG-WRN in wild-type C33a cells (Fig. 3B). E1 contributes to the reduction of WRN levels observed with the E1-E2 replication complex (Fig. 4A and B). There is an elevated level of E2 in the presence of E1 due to enhanced stability, as we have previously reported (75). Coimmunoprecipitation experiments demonstrate that FLAG-WRN and E1 exist in the same cellular complex (Fig. 4C), while E2 does not (Fig. 4A and B). As E1 can activate the DDR by itself and results in reduced levels of FLAG-WRN, we next tested whether E1 regulates the stability of FLAG-WRN. Cycloheximde (CHX) chase experiments demonstrated that FLAG-WRN is stable in both C33a cells and C33a SIRT1^–/–^ clone 1 cells (Fig. 4D and E, respectively). However, in the presence of the E1-E2 replication complex, FLAG-WRN is destabilized in wild-type C33a cells (Fig. 4F). However, in the absence of SIRT1, FLAG-WRN is stabilized (Fig. 4G) and E2 is also stabilized in C33a SIRT1^–/–^ clone 1 cells, as previously reported (41). The levels of WRN expression were quantified, and summaries are shown in Fig. S4A to D. The E1 protein retained the ability to complex with FLAG-WRN in the absence of SIRT1 (Fig. S4E).

10.1128/mBio.00263-19.4 FIG S4 (A) This is a quantitation of Fig. 4D of repeat experiments. There is no significant difference in FLAG-WRN levels at any time point following cycoheximide (CHX) addition in wild-type C33a cells (standard error bars are shown). (B) This is a quantitation of Fig. 4E of repeat experiments. There is no significant difference in FLAG-WRN levels at any time point following CHX addition in C33a SIRT1^–/–^ clone 1 cells (standard error bars are shown). Panels A and B depict the results of two independent experiments. (C) This is a quantitation of Fig. 4F of repeat experiments. There is a significant decrease (*) in FLAG-WRN levels in the presence of the E1-E2 replication complex following CHX addition for 12 h in C33a wild-type cells (*P* value was less than 0.05; standard error bars are shown). (D) This is a quantitation of Fig. 4G. There is no significant difference in FLAG-WRN levels at any time point following CHX addition to C33a SIRT1^–/–^ clone 1 cells when the E1-E2 replication complex is coexpressed. The results of this quantitation for the blot in panels C and D are represented graphically and represent the summary of results from three independent experiments (standard error bars are shown). (E) E1 interaction with FLAG-WRN is retained in the absence of SIRT1. E1-E2-pOri plasmids were transfected into C33a (lanes 1 to 3) and C33a SIRT1^–/–^ clone 1 (lanes 4 to 6) cells, and the expression of E1 and FLAG-WRN was confirmed (upper panel). These extracts were then used in an immunoprecipitation with an HA antibody that recognizes the HA-tagged E1 protein. The lower panel demonstrates that E1 interacts with FLAG-WRN in C33a (lower panel, lane 3) and in C33a SIRT1^–/–^ clone 1 (lower panel, lane 6) cells. Download FIG S4, TIF file, 8.1 MB. Copyright © 2019 Das et al. 2019 Das et al. This content is distributed under the terms of the Creative Commons Attribution 4.0 International license.

**FIG 5:**
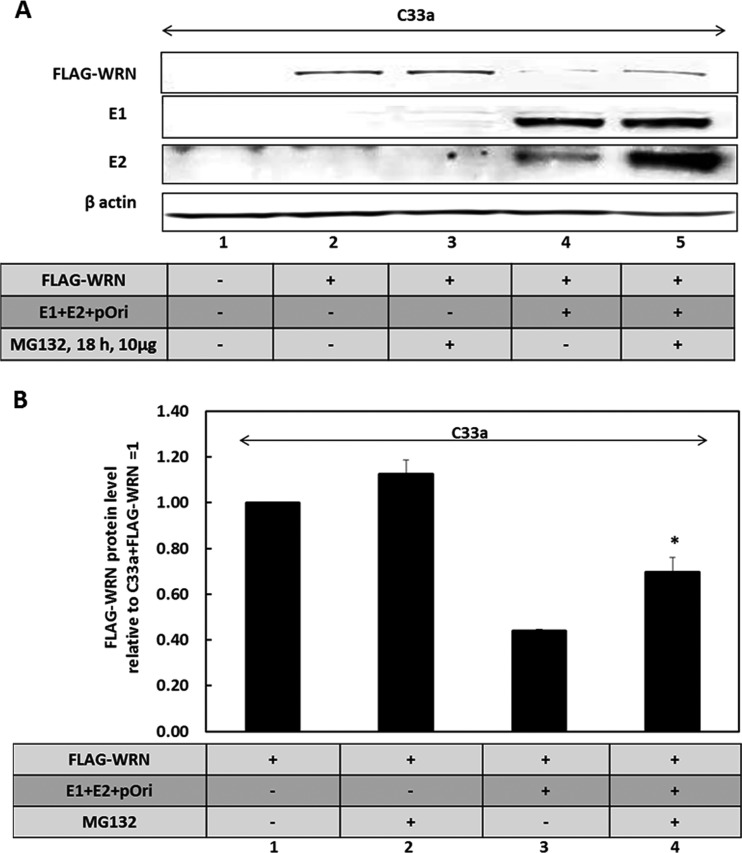
WRN protein turnover is enhanced via the proteasome in the presence of E1. (A) C33a cells were transfected with the indicated plasmids and treated with the proteasome inhibitor MG132 for 18 h prior to cell harvest. Protein extracts were then prepared, and Western blotting for the indicated proteins was carried out. MG132 stabilizes both E2 and FLAG-WRN (compare the levels in lane 5 with those in lane 4). (B) The experiment in panel J was repeated three times, and the results were quantitated and graphed on a histogram. There is a significant increase (*) in FLAG-WRN in the presence of MG132 when the E1-E2 replication complex is present (*P* value was less than 0.05; standard error bars are shown).

The results suggest that the activation of the DDR by E1 (as we and others have demonstrated previously) stimulates SIRT1 to deacetylate WRN. This deacetylation contributes to the destabilization of the WRN protein when the DDR is activated, and previous studies have demonstrated that activation of the DDR results in promotion of WRN degradation (76). Acetylation of WRN prevents ubiquitination and therefore inhibits its degradation via the proteasome (57); therefore, depletion of SIRT1 protects the E1-mediated degradation of FLAG-WRN, but it also blocks the recruitment of WRN to the E1-E2-replicating DNA due to the elevated acetylation status of the WRN protein. The addition of MG132 partially restores WRN expression levels in the presence of E1 (Fig. 5A and B), suggesting that activation of the DDR by E1 promotes degradation of WRN via the proteasome, similarly to exogenous agents that activate the DDR (76). The E2 protein was stabilized by the addition of MG132, and we have demonstrated previously that the turnover of this protein is regulated via the proteasome (77).

**FIG 6:**
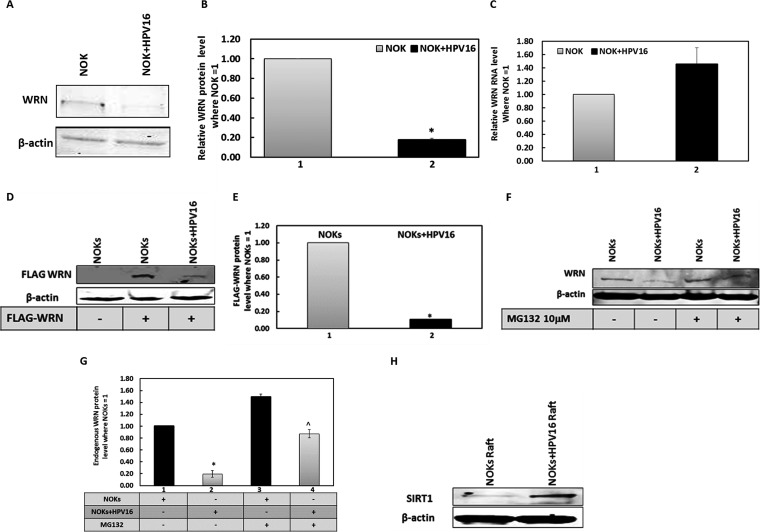
WRN protein turnover is regulated by the entire HPV16 genome in nontransformed keratinocytes, similarly to E1-E2 replication in C33a cells. (A) NOKs and NOKs plus HPV16 (cells that contain episomal HPV16 genomes and support late stages of the viral life cycle [78]) were blotted for endogenous WRN protein levels. (B) Duplicate experiments of that shown in panel A were quantitated, and there is a significant decrease in WRN protein levels in the presence of HPV16 (*, *P* value was less than 0.05; standard error bars are shown). (C) This reduction is not due to a reduction in WRN RNA levels. Results from an average of three independent experiments are shown from reverse transcriptase quantitative PCR, and there is no significant difference in WRN RNA in the absence or presence of HPV16 (standard error bars are shown). (D) NOKs and NOKs plus HPV16 were transiently transfected with the FLAG-WRN expression vector, and 48 h later, protein extracts were prepared and FLAG Western blotting was carried out. (E) Duplicate experiments of that shown in panel D were quantitated, and there is a significant decrease in FLAG-WRN levels in the presence of HPV16 (*; *P* value was less than 0.05; standard error bars are shown). (F) NOKs and NOKs plus HPV16 were treated with MG132 for 18 h prior to the preparation of protein extracts and Western blotting for endogenous WRN. (G) The results of duplicate experiments of that shown in panel F were quantitated, and there is a significant increase in WRN protein levels in NOKs plus HPV16 but not NOKs following MG132 treatment (*; *P* value was less than 0.05; standard error bars are shown). (H) Protein extracts were prepared from NOKs and NOKs plus HPV16 cells that had been subjected to organotypic rafting. HPV16 induces levels of SIRT1 in the NOKs, as evidenced by the large increase in SIRT1 protein detection.

To date, the results have depended upon our model systems, which involve overexpression of the E1 and E2 proteins. We next wanted to determine whether in a model of HPV16 infection WRN levels were reduced by HPV16. To do this, we investigated WRN levels in oral keratinocytes that contain the HPV16 genome and support late stages of the viral life cycle (78). Normal oral keratinocytes (NOKs) were derived from oral epithelium and immortalized using telomerase (79), and we added the HPV16 genome to these cells and demonstrated a host transcriptional reprogramming, an activation of the DDR, and the expression of several viral markers demonstrating late stages of the viral life cycle in these cells (78). Western blots demonstrate that the presence of HPV16 in the NOKs results in a decrease in WRN levels (Fig. 6A); this was repeated and quantitated (Fig. 6B). This reduction was not due to a change in WRN RNA levels (Fig. 6C) and suggests that the replication DDR signal generated by the entire HPV16 genome reduces WRN levels in oral keratinocytes. Next, we demonstrated that FLAG-WRN is preferentially downregulated in NOKs plus HPV16 versus NOKs (Fig. 6D); this was repeated and quantitated (Fig. 6E). To demonstrate that WRN levels are downregulated by HPV16 in NOKs, we added MG132 (Fig. 6F) and partially recovered the expression of WRN in the NOKs plus HPV16 cells; this was repeated and quantitated (Fig. 6G). Both of these results are similar to those that we observed in C33a cells, where FLAG-WRN levels are reduced by E1-E2-replicating DNA via the proteasome. Therefore, the targeting of WRN by E1-E2 replication in C33a cells is mimicked in cells that contain an HPV16 genome and support the late stages of the viral life cycle. We also investigated the levels of SIRT1 and WRN during the viral life cycle by analyzing their levels in protein extracts from organo-typic raft cultures. SIRT1 levels are dramatically increased by HPV16 in differentiated cells, as there are high levels in NOKs plus HPV16 and low levels in NOKs (Fig. 6H). This supports our model that the virus increases SIRT1 activity to promote the viral life cycle and agrees with the work of others (56). We could not detect WRN in the extracts from the differentiated raft tissue, perhaps because the protein was degraded during the extraction process.

### Functional interaction between SIRT1, WRN, and E1 during E1-E2-mediated DNA replication.

**FIG 7:**
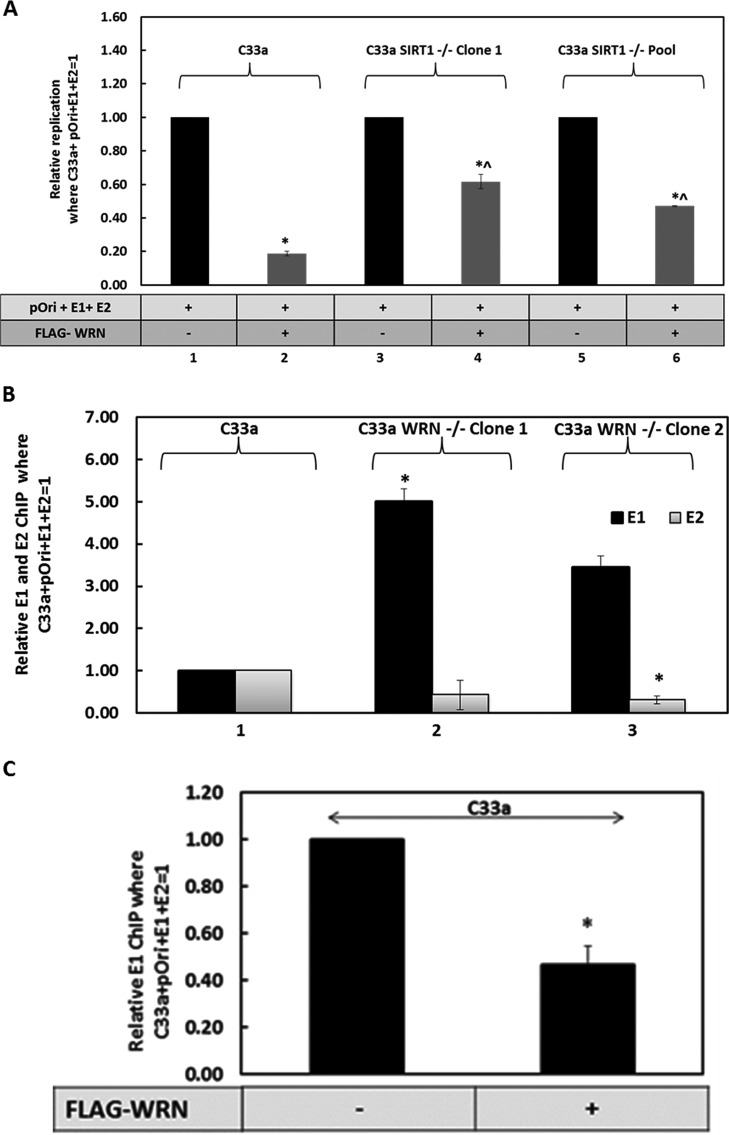
WRN regulates recruitment of E1 to E1-E2-replicating DNA. (A) WRN significantly represses E1-E2 DNA replication irrespective of SIRT1 status (*), but this repression is significantly less in the absence of WRN (^) (*P* values were less than 0.05 in all cases; standard error bars are shown). Results represent a summary of the results from at least 3 independent experiments, and standard error bars are shown. (B) In the absence of WRN, there are elevated levels of E1 on E1-E2-replicating DNA, as determined using ChIP. This reaches significance (*) in C33a WRN^–/–^ clone 1 (*P* values were less than 0.05) but just fails to reach significance in clone 2 (lane 3), even though there is increased E1 detected. There is a reduction in E2 on E1-E2-replicating DNA in the absence of WRN, and this reaches significance in C33a WRN^–/–^ clone 2 (*; *P* value was less than 0.05), with the same downward trend in C33a WRN^–/–^ clone 1. Results are a summary of the results of at least 3 independent experiments, and standard error bars are shown. (C) When FLAG-WRN is coexpressed with the E1-E2-pOri replication complex, there is a significant decrease in E1 binding to the pOri plasmid. *, *P* value was less than 0.05 (a standard error bar is also shown). This is a summary of the results of three independent experiments.

The overexpression of SIRT1 in C33a cells does not alter E1-E2 DNA replication properties, although removal of SIRT1 does boost this replication (41). The proposed mechanism of this increase in replication is an increased acetylation and stabilization of the E2 protein that enhances replication (41). C33a cells already express a high level of SIRT1, and therefore, presumably, increasing the levels from exogenous plasmids has no effect on the overall function of SIRT1 in E1-E2 replication. However, overexpression of WRN can repress E1-E2 DNA replication (Fig. 2B). Both E1 and WRN can bind to DNA, and both have 3′-to-5′ helicase activity; we investigated whether E1 and WRN compete for the E1-E2-replicating DNA. Such competition would result in elevation of E1 levels on the replicating DNA in the absence of WRN and would also explain why overexpression of WRN represses E1-E2 replication. If this mechanism is true, WRN repression of E1-E2 DNA replication should be reduced in the absence of SIRT1 due to the failure of the acetylated WRN to bind to replicating DNA. This is indeed the case (Fig. 7A). In wild-type C33a cells, overexpression of WRN substantially represses E1-E2 replication (Fig. 7A, compare lane 2 with lane 1), but in the absence or depletion of SIRT1 levels, there is a reduction in this repression (compare lanes 4 and 6 with lane 2). This is reflective of a reduced recruitment of FLAG-WRN to E1-E2-replicating DNA in the absence of WRN (Fig. 3E). The results are presented relative to the levels in wild-type C33a cells, with the E1-E2 replication complex equaling 1. Figure S5A presents the control for these experiments.

10.1128/mBio.00263-19.5 FIG S5 (A) This is a control figure for Fig. 7A. The results shown are with wild-type C33a cells with and without the E1 and E2 proteins and demonstrate the large increase in signal when the replication proteins are there. This was then used as 1 to standardize the experimental results in Fig. 7A. There is a significant decrease in signal in the absence of the E1 and E2 proteins (*; *P* value was less than 0.05). Results represent a summary of the results from at least 3 independent experiments, and standard error bars are shown. (B) This is a control figure for Fig. 7B. This figure demonstrates that the signal obtained in the ChIP experiments when no E1 or E2 is expressed is negligible. This is a signal generated by the HA and E2 antibodies to detect HA-tagged E1 and E2, respectively. There is significantly less signal (*) in the absence of the E1 and E2 proteins; standard error bars are shown. The expression of either replication protein by itself also does not enhance the signal (11, 41). (C) This is a control figure for Fig. 7B. This figure demonstrates the signal obtained with a control antibody (rabbit serum); there is no increase in signal with the presence of E1 and E2, and this represents a background signal. This demonstrates the specificity of the E1 and E2 signals in Fig. 7B. The results in both panel B and panel C represent a summary of the results of at least 3 independent experiments; standard error bars are shown. (D) This is a control figure for Fig. 7C. This figure demonstrates the signal obtained with a control antibody (rabbit serum). There is no increase in signal with the presence of E1 and E2, and this represents a background signal. This demonstrates the specificity of the E1 signal in Fig. 7C. (E) This is a control figure for Fig. 7C. This figure demonstrates that the signal obtained in the ChIP experiments when no E1 or E2 was expressed is negligible. This is a signal generated by the HA antibody to detect HA-tagged E1. There is significantly less signal (*) in the absence of the E1 and E2 proteins; standard error bars are shown. Expression of either replication protein by itself also does not enhance the signal (D. Das, N. Smith, X. Wang, and I. M. Morgan, J Virol 91:e00102-17, 2017, https://doi.org/10.1128/JVI.00102-17; E. J. Gauson, M. M. Donaldson, E. S. Dornan, X. Want, et al., J Virol 89:17684–17699, 2015, https://doi.org/10.1128/JVI.00335-15). The results in both panels D and E represent a summary of the results of at least 3 independent experiments; standard error bars are shown. Download FIG S5, TIF file, 0.1 MB. Copyright © 2019 Das et al. 2019 Das et al. This content is distributed under the terms of the Creative Commons Attribution 4.0 International license.

If there is competition between E1 and WRN for the E1-E2-replicating DNA, then we would expect elevated levels of E1 on the DNA in the absence of WRN. To test this, we used our CRISPR/Cas9 WRN knockout C33a cells. The results demonstrate that in the absence of WRN, there is indeed an elevated level of E1 on the replicating DNA (Fig. 7B). The controls for these ChIP experiments are shown in Fig. S5B and S5C. There is no change in the levels of the E1 and E2 proteins in the absence of WRN (Fig. 2C); therefore, there is a difference in levels of recruitment to the replicating DNA. This suggests that E1 and WRN are in competition for replicating DNA and that in the absence of WRN, the E1 protein has an enhanced ability to bind to the replicating DNA. This would explain the increase in E1-E2 replication observed in the absence of WRN (Fig. 2B). Finally, if our model is correct, overexpression of FLAG-WRN in wild-type C33a cells should compete with E1 for the replicating DNA, and this is indeed what we observed (Fig. 7C). The controls for these ChIP assays are presented in Fig. S5D and S5E.

## DISCUSSION

There is an intricate interaction between HPV and the DDR that promotes the viral life cycle (43–47, 49, 50); therefore, efficient targeting of the HPV-induced DDR offers therapeutic opportunities. Here, we demonstrate that lack of SIRT1 results in elevated and mutagenic E1-E2 DNA replication. Contributing to this mutagenic replication is a failure to recruit WRN to the E1-E2-replicating DNA due to an enhanced acetylation that prevents the interaction of WRN with the E1-E2-replicating DNA, even though there are enhanced levels of WRN in the absence of SIRT1. Deletion of WRN from cells has a E1-E2 replication phenotype identical to that of deletion of SIRT1, elevated replication with an enhanced mutation frequency. The elevation of replication in the absence of SIRT1 is likely due to an enhanced stability of E2 in the absence of SIRT1 that is mediated by an elevated acetylation and stability of the E2 protein (41), while for WRN it is likely related to the increased recruitment of the E1 replication factor to the replicating DNA in the absence of WRN. There is no change in the levels of the viral proteins in the absence of WRN. Both E1 and WRN have 3′-to-5′ helicase activity (WRN also has a 3′-to-5′ exonuclease activity that contributes to its DNA repair function), and therefore it is possible that both proteins compete for binding to the E1-E2-replicating DNA.

The results present the following model. Following infection, the E1-E2 proteins (along with the other E viral proteins) are expressed and replication is initiated. This replication activates the DDR; E1 can do this by itself, and E1 and E2 can do this together (48, 71–74). Notably, E1-E2 replication is not arrested in the presence of an active DDR (48, 80). At this early stage of the viral life cycle, the virus has to increase its genome copy number to around 20 to 50 genomes per cell; therefore, there is the potential for replication stress on the viral genome during repeated initiation of replication, resulting in replication fork clashes (52); this replication stress and the formation of aberrant DNA structures activates the DDR. There is then the recruitment of host HR factors to the viral genome, and it is proposed that this recruitment results in HPV employing an HR mechanism of DNA replication (51). HR allows the virus to resolve these aberrant DNA structures and clashing replication forks to enable successful amplification of the viral genome. The activation of the DDR then stimulates SIRT1 activity to deacetylate substrates that promote HR and efficient repair of damaged DNA (38–40, 42, 53, 55, 59, 67, 81–84). One of these substrates is WRN, and its deacetylation promotes the interaction of WRN with damaged DNA (57, 58, 68). This is precisely what we observed with E1-E2-replicating DNA; in the absence of SIRT1, there are elevated levels of WRN acetylation, and this acetylated DNA has a reduced capacity for interaction with the replicating DNA, promoting mutagenic replication. It is known that the WRN protein is involved in promoting high-fidelity replication and has proposed roles in repairing stalled replication forks and contributing to the HR process, perhaps by assisting with resection of double-stranded DNA using its 3′-to-5′ exonuclease activity (61–64, 85–91). The precise roles of the enzymatic activity of WRN in the DNA repair process is unclear, and the E1-E2 replication system offers a unique opportunity to determine the contribution of these activities to the maintenance of genomic integrity as complementation with wild-type WRN restores the fidelity of E1-E2 replication in the WRN knockout cells.

Activation of the DDR stimulates WRN activity, and subsequently, levels decrease over a 12-h period following ATR (ataxia telangiectasia and Rad3 related) phosphorylation; WRN is turned over via the proteasome (76). HPV replication stimulates ATR activity (44, 92), and it is noticeable that in the presence of E1-E2 replication, levels of WRN are reduced. This reduction is partially reversed in the absence of SIRT1, as WRN is acetylated on lysine residues that are also targeted for ubiquitination; therefore, elevated acetylation in the absence of SIRT1 protects WRN from degradation (57). This is precisely what we observed in our results; FLAG-WRN levels are reduced in the presence of the E1-E2 replication complex, but in the absence of SIRT1, there are elevated acetylation levels of WRN and an increased level of the protein. We demonstrate that in wild-type cells, E1-E2 replication reduces the half-life of the WRN protein, and this reduction is abrogated in the absence of SIRT1. We also demonstrate that MG132 treatment can partially restore WRN levels in the presence of the E1-E2 replication complex. Overall, the results suggest that E1-E2 activation of the DDR promotes ATR phosphorylation of WRN to promote its degradation via the proteasome. However, it is clear that not all of WRN is degraded, as WRN is important for promoting the fidelity of E1-E2 replication. The virus seems to balance the levels of WRN; activation of the DDR targets the protein for degradation via the proteasome, and this requires SIRT1 deacetylation. However, it retains an active level of WRN, which promotes the fidelity of replication, as a total absence of WRN results in mutagenic replication.

What does this mean for the viral life cycle? It is clear that high-risk HPV containing keratinocytes have an active DDR yet can still undergo a cell cycle (46, 78); therefore, the DDR is different from that stimulated by an external DNA-damaging agent that promotes cell cycle arrest and DNA damage repair, followed by a restart of DNA replication and reentry into the cell cycle. It remains to be fully elucidated how virally infected cells retain an ability to have an active DDR and an ongoing cell cycle. As WRN is crucial to replication fork arrest and repair of DNA, it is possible that the reduced levels of WRN stimulated by E1 and E2 (which are also observed in our oral-keratinocyte model of HPV16) are required for the infected cell to cycle in the presence of the DDR. Reduced levels of SIRT1 block the HPV31 life cycle (56), and failure to recruit WRN to the viral DNA in the absence of SIRT1 might play a role in this. However, the results here also present a word of caution about targeting SIRT1 therapeutically to intervene in high-risk HPV life cycles to block infection; manipulation of SIRT1 might result in elevated viral mutagenic replication that promotes double-strand DNA breaks, providing substrates for viral integration. Tumors with integrated genomes have a more aggressive phenotype.

What does this mean for therapeutic approaches to HPV diseases? Recently, it has been demonstrated that the majority of HPV16-positive head and neck cancers retain an episomal viral genome replicating in an E1-E2-dependent manner (93–96); therefore, direct targeting of HPV replication offers therapeutic opportunities. Currently, we investigate pathway manipulation (including the DDR) that could stabilize the WRN protein in high-risk HPV-positive cells; such elevation would block E1-E2 replication. This would reduce the viral genome copy number in cancer cells and might contribute to therapeutic targeting of HPV-positive cancers with episomal viral genomes. In addition, cells that lack WRN have an increased sensitivity to certain DNA-damaging drugs, including camptothecin. It would be interesting to test the difference in the responses of HPV16-positive and -negative cancers to this drug, and we are currently developing patient-derived xenograft models for this purpose.

Overall, the results demonstrate that SIRT1 and WRN contribute to E1-E2 replication control and fidelity and that they likely act in a coordinated fashion. Future studies will focus on gaining further insights into the mechanisms that these proteins use to regulate E1-E2 replication and high-risk-HPV life cycles, with a view to determining novel ways to target viral replication for therapeutic gain. One final comment is that this downregulation of WRN would also result in an increased vulnerability for the host genome to mutagenesis; therefore, this is also a novel mechanism that might contribute to high-risk-HPV oncogenesis.

## MATERIALS AND METHODS

### Cell line, plasmids, and reagents.

C33a cells (catalog number HTB-31) were obtained from the American Type Culture Collection and were grown in Dulbecco modified Eagle medium (DMEM) with 10% fetal bovine serum (FBS) in a humidified CO_2_ incubator in 5% CO_2_ at 37°C. SIRT1-depleted C33a cells have been described previously (41). Clonal cell lines containing the HPV16 genome were generated from normal oral keratinocytes (NOKs), as previously described (78). These cells were cultured alongside parental NOKs for all comparisons. NOKs and NOKs plus HPV16 cells were grown in K-SFM (Invitrogen) with a 1% (vol/vol) penicillin-streptomycin mixture (Thermo Fisher Scientific) containing 4 μg/ml hygromycin B (Millipore Sigma) at 37°C in a 5% CO_2_-95% air atmosphere and passaged every 3 to 4 days. NOKs plus HPV16 were grown in the same medium but also containing 150 μg/ml G418 (Thermo Fisher Scientific). The HPV16-E2 (97), hemagglutinin-E1 (HA-E1) (72), pOri (69), and pOri-Lacz (66) plasmids have been described previously. For WRN knockout CRISPR, WRN double-nickase plasmid (h) (catalog number sc-401860-NIC) was purchased from Santa Cruz. C33a WRN^–/–^ clone 1 and clone 2 were generated as described for the SIRT1 knockout cells (41). The double-nickase plasmid consists of a pair of plasmids, each of which encodes a Cas9 nuclease with a D10A mutation and a target-specific 20-nucleotide guide RNA designed to knock out particular gene expression with greater specificity than a single CRISPR/Cas9 knockout counterpart. The FLAG–wild-type SIRT1 (catalog number 1791), FLAG-MT SIRT1 (H363Y) (catalog number 1792) and MYC–wild-type WRN (catalog number pMM290) plasmids were purchased from Addgene. The FLAG-WRN expression plasmid has been described previously (91). Cycloheximide (catalog number 97064-724) was purchased from VWR (USA). MG132 (catalog number C2211-5MG) was purchased from Sigma (USA).

### Organotypic raft culture.

NOKs and NOKs plus HPV16 cells were differentiated via organotypic raft culture as described previously (78, 98). Briefly, cells were seeded onto type 1 collagen matrices containing J2 3T3 fibroblast feeder cells. Cells were then grown to confluence atop the collagen matrices, which were then lifted onto wire grids and cultured in cell culture dishes at the air-liquid interface, with medium replacement on alternate days. Following 13 days of culture, rafted samples were fixed with formaldehyde (4%, vol/vol) and embedded in paraffin blocks. Multiple 4-μm sections were cut from each sample. Sections were stained with hematoxylin and eosin (H&E) and others prepared for immunofluorescent staining as described previously. Fixing and embedding services in support of the research project were generated by the VCU Massey Cancer Center Cancer Mouse Model Shared Resource, supported, in part, with funding from the NIH (NCI Cancer Center support grant P30 CA016059).

### Western blotting.

Cells were harvested and proteins extracted with lysis buffer (0.5% Nonidet P-40 [NP-40], 50 mM Tris, pH 7.8, 150 mM NaCl with protease inhibitor cocktail and phosphatase inhibitor), and Western blotting was carried out as described previously (41). Approximately 50 µg of protein was run on 4%-to-12% gradient gel, after which it was transferred onto a nitrocellulose membrane. The membrane was blocked with Odyssey blocking buffer and then incubated with the corresponding primary antibodies. Imaging was done using the Odyssey Li-Cor imaging system. The images were quantified via Image Studio Lite version 5.2 software and are represented as histograms.

### ChIP.

Cells after being plated at a density of 5 × 10^5^ were transfected with 1 μg each of pOri, E1 plasmid, and E2 plasmid using the CaPO4 precipitation method. Forty-eight hours posttransfection, the cells were harvested by being scraped and processed for chromatin as described previously (41). Chromatin concentration was determined with a NanoDrop spectrophotometer. Approximately 100 µg of chromatin from each sample was used for the experiment. A/G magnetic beads were used to pull down the antibody-chromatin complex. To show antibody specificity, each of the samples were pulled down with the rabbit isotype control shown in the figures in the supplemental material. The immunoprecipitated chromatin was processed for quantitative PCR (qPCR), and a pOri primer was used to measure the levels of immunoprecipitation of the chromatin.

### Replication assay.

Cells were plated in a 100-mm^2^ tissue culture disc and transfected with 10 ng pOri, 1 µg E1 plasmid, and 10 ng E2 plasmid using CaPO_4_ precipitation (41). Forty-eight hours posttransfection, the cells were washed with 1× phosphate-buffered saline (PBS) and then harvested using Hirt solution (10 mM EDTA, 0.5% SDS), and the samples were processed for qPCR as described previously (69).

### DNA mutagenesis analysis.

DNA was harvested as described for the replication assay, and the samples were digested with DpnI to remove the input DNA and then extracted with phenol-chloroform-isoamyl alcohol (25:24:1). DNA was precipitated with ethanol and was resuspended in 150 µl of 10% glycerol. Seventy-five microliters of the DNA was electroporated into DH10B bacteria and plated on 100 µg/ml X-gal (5-bromo-4-chloro-3-indolyl-β-d-galactopyranoside) lysogeny broth (LB) agar with kanamycin selection (66).

### IP.

Two hundred micrograms of protein lysate from each sample was used for the pulldown, and the volume was made up to 300 µl using lysis buffer. Two micrograms of antibody was used for the pulldown as described previously (41). The following day, protein A-Sepharose bead slurry was added to each sample, and samples were incubated on a rotor at 4°C for 5 h. The protein-bead mixture was then washed and processed for Western blotting (41).

### CHX time chase.

Forty-eight hours posttransfection, 100 μg/ml cycloheximide (CHX)-containing medium was added to each plate for the time points specified in the figures. After incubation, the cells were harvested and processed for Western blotting.

### Proteasomal degradation.

The cells were pretreated with 10 µg of MG132 for 18 h before being harvested and processed for Western blotting.

### RNA assay.

The SV total RNA isolation system kit (Promega) was used to isolate RNA from cells. A high-capacity cDNA reverse-transcription kit from Invitrogen was used to synthesize cDNA, which was processed for qPCR.

### Immunoflourescence.

Cells were grown on coverslips to ∼50% confluence and transfected with the respective plasmid. Forty-eight hours posttransfection, the cells were fixed with methanol and washed with PBS repeatedly. Cells were made permeable using 0.2% Triton X-100 in PBS for 15 min at 4°C and then washed with PBS. Cells were then incubated with the respective primary and secondary antibody at 4°C in a humified chamber sequentially. The coverslips were then washed and stained with DAPI (4′,6-diamidino-2-phenylindole) and mounted on slides using Vectashield mounting medium (ThermoFisher catalog number NC9265087). Images were taken using a Zeiss LSM 700 confocal laser-scanning microscope and analyzed using ZEN lite software (48).

### Statistical analysis.

We employed a two-tailed Student *t* test in which a *P* of <0.05 (*) and a *P* of <0.05 (^) were considered to be statistically significant.

## References

[1] Zur Hausen H 2009 Papillomaviruses in the causation of human cancers—a brief historical account. Virology 384:260–265. doi:10.1016/j.virol.2008.11.046. 19135222

[2] Marur S, D'Souza G, Westra WH, Forastiere AA 2010 HPV-associated head and neck cancer: a virus-related cancer epidemic. Lancet Oncol 11:781–789. doi:10.1016/S1470-2045(10)70017-6. 20451455 PMC5242182

[3] Stanley MA, Pett MR, Coleman N 2007 HPV: from infection to cancer. Biochem Soc Trans 35:1456–1460. doi:10.1042/BST0351456. 18031245

[4] Thierry F 2009 Transcriptional regulation of the papillomavirus oncogenes by cellular and viral transcription factors in cervical carcinoma. Virology 384:375–379. doi:10.1016/j.virol.2008.11.014. 19064276

[5] Stanley MA 2012 Epithelial cell responses to infection with human papillomavirus. Clin Microbiol Rev 25:215–222. doi:10.1128/CMR.05028-11. 22491770 PMC3346303

[6] Moody CA, Laimins LA 2010 Human papillomavirus oncoproteins: pathways to transformation. Nat Rev Cancer 10:550–560. doi:10.1038/nrc2886. 20592731

[7] Masterson PJ, Stanley MA, Lewis AP, Romanos MA 1998 A C-terminal helicase domain of the human papillomavirus E1 protein binds E2 and the DNA polymerase alpha-primase p68 subunit. J Virol 72:7407–7419. 9696837 10.1128/jvi.72.9.7407-7419.1998 PMC109968

[8] Clower RV, Fisk JC, Melendy T 2006 Papillomavirus E1 protein binds to and stimulates human topoisomerase I. J Virol 80:1584–1587. doi:10.1128/JVI.80.3.1584-1587.2006. 16415033 PMC1346965

[9] Loo YM, Melendy T 2004 Recruitment of replication protein A by the papillomavirus E1 protein and modulation by single-stranded DNA. J Virol 78:1605–1615. doi:10.1128/JVI.78.4.1605-1615.2004. 14747526 PMC369418

[10] Melendy T, Sedman J, Stenlund A 1995 Cellular factors required for papillomavirus DNA replication. J Virol 69:7857–7867. 7494298 10.1128/jvi.69.12.7857-7867.1995 PMC189730

[11] Gauson EJ, Donaldson MM, Dornan ES, Wang X, Bristol M, Bodily JM, Morgan IM 2015 Evidence supporting a role for TopBP1 and Brd4 in the initiation but not continuation of human papillomavirus 16 E1/E2-mediated DNA replication. J Virol 89:17684–17699. doi:10.1128/JVI.00335-15. PMC4403487 25694599

[12] Donaldson MM, Mackintosh LJ, Bodily JM, Dornan ES, Laimins LA, Morgan IM 2012 An interaction between human papillomavirus 16 E2 and TopBP1 is required for optimum viral DNA replication and episomal genome establishment. J Virol 86:12806–12815. doi:10.1128/JVI.01002-12. 22973044 PMC3497701

[13] Boner W, Taylor ER, Tsirimonaki E, Yamane K, Campo MS, Morgan IM 2002 A functional interaction between the human papillomavirus 16 transcription/replication factor E2 and the DNA damage response protein TopBP1. J Biol Chem 277:22297–22303. doi:10.1074/jbc.M202163200. 11934899

[14] McBride AA 2013 The papillomavirus E2 proteins. Virology 445:57–79. doi:10.1016/j.virol.2013.06.006. 23849793 PMC3783563

[15] Bergvall M, Melendy T, Archambault J 2013 The E1 proteins. Virology 445:35–56. doi:10.1016/j.virol.2013.07.020. 24029589 PMC3811109

[16] Kanginakudru S, DeSmet M, Thomas Y, Morgan IM, Androphy EJ 2015 Levels of the E2 interacting protein TopBP1 modulate papillomavirus maintenance stage replication. Virology 478:129–135. doi:10.1016/j.virol.2015.01.011. 25666521 PMC4361275

[17] Zhou Z-W, Liu C, Li T-L, Bruhn C, Krueger A, Min W, Wang Z-Q, Carr AM 2013 An essential function for the ATR-activation-domain (AAD) of TopBP1 in mouse development and cellular senescence. PLoS Genet 9:e1003702. doi:10.1371/journal.pgen.1003702. 23950734 PMC3738440

[18] Morishima K, Sakamoto S, Kobayashi J, Izumi H, Suda T, Matsumoto Y, Tauchi H, Ide H, Komatsu K, Matsuura S 2007 TopBP1 associates with NBS1 and is involved in homologous recombination repair. Biochem Biophys Res Commun 362:872–879. doi:10.1016/j.bbrc.2007.08.086. 17765870

[19] Yoo HY, Kumagai A, Shevchenko A, Shevchenko A, Dunphy WG 2007 Ataxia-telangiectasia mutated (ATM)-dependent activation of ATR occurs through phosphorylation of TopBP1 by ATM. J Biol Chem 282:17501–17506. doi:10.1074/jbc.M701770200. 17446169

[20] Kumagai A, Lee J, Yoo HY, Dunphy WG 2006 TopBP1 activates the ATR-ATRIP complex. Cell 124:943–955. doi:10.1016/j.cell.2005.12.041. 16530042

[21] Yoo HY, Kumagai A, Shevchenko A, Shevchenko A, Dunphy WG 2009 The Mre11-Rad50-Nbs1 complex mediates activation of TopBP1 by ATM. Mol Biol Cell 20:2351–2360. doi:10.1091/mbc.e08-12-1190. 19279141 PMC2675615

[22] Garcia V, Furuya K, Carr AM 2005 Identification and functional analysis of TopBP1 and its homologs. DNA Repair (Amst) 4:1227–1239. doi:10.1016/j.dnarep.2005.04.001. 15897014

[23] Beuzer P, Quivy J-P, Almouzni GV 2014 Establishment of a replication fork barrier following induction of DNA binding in mammalian cells. Cell Cycle 13:1607–1616. doi:10.4161/cc.28627. 24675882 PMC4050166

[24] Boos D, Yekezare M, Diffley JFX 2013 Identification of a heteromeric complex that promotes DNA replication origin firing in human cells. Science 340:981–984. doi:10.1126/science.1237448. 23704573

[25] Lee J, Dunphy WG 2013 The Mre11-Rad50-Nbs1 (MRN) complex has a specific role in the activation of Chk1 in response to stalled replication forks. Mol Biol Cell 24:1343–1353. doi:10.1091/mbc.e13-01-0025. 23468519 PMC3639046

[26] Leung CC, Sun L, Gong Z, Burkat M, Edwards R, Assmus M, Chen J, Glover JN 2013 Structural insights into recognition of MDC1 by TopBP1 in DNA replication checkpoint control. Structure 21:1450–1459. doi:10.1016/j.str.2013.06.015. 23891287 PMC3760280

[27] Tanaka S, Komeda Y, Umemori T, Kubota Y, Takisawa H, Araki H 2013 Efficient initiation of DNA replication in eukaryotes requires Dpb11/TopBP1-GINS interaction. Mol Cell Biol 33:2614–2622. doi:10.1128/MCB.00431-13. 23629628 PMC3700120

[28] Boos D, Sanchez-Pulido L, Rappas M, Pearl LH, Oliver AW, Ponting CP, Diffley JFX 2011 Regulation of DNA replication through Sld3-Dpb11 interaction is conserved from yeast to humans. Curr Biol 21:1152–1157. doi:10.1016/j.cub.2011.05.057. 21700459

[29] Kumagai A, Shevchenko A, Shevchenko A, Dunphy WG 2011 Direct regulation of Treslin by cyclin-dependent kinase is essential for the onset of DNA replication. J Cell Biol 193:995–1007. doi:10.1083/jcb.201102003. 21646402 PMC3115804

[30] Navadgi-Patil V, Kumar S, Burgers PM 2011 The unstructured C-terminal tail of yeast Dpb11 (Human TopBP1) protein is dispensable for DNA replication and the S phase checkpoint but required for the G 2/M checkpoint. J Biol Chem 286:40999–41007. doi:10.1074/jbc.M111.283994. 21956112 PMC3220456

[31] Vol CB, Mueller AC, Keaton MA, Dutta A 2011 DNA replication: mammalian Treslin TopBP1 interaction mirrors yeast Sld3 Dpb11. Curr Biol 21:R638–R640. doi:10.1016/j.cub.2011.07.004. 21855008 PMC3523092

[32] Balestrini A, Cosentino C, Errico A, Garner E, Costanzo V 2010 GEMC1 is a TopBP1-interacting protein required for chromosomal DNA replication. Nat Cell Biol 12:484–491. doi:10.1038/ncb2050. 20383140 PMC2875115

[33] Kumagai A, Shevchenko A, Shevchenko A, Dunphy WG 2010 Treslin collaborates with TopBP1 in triggering the initiation of DNA replication. Cell 140:349–359. doi:10.1016/j.cell.2009.12.049. 20116089 PMC2857569

[34] Liu K, Bellam N, Lin H-Y, Wang B, Stockard CR, Grizzle WE, Lin W-C 2009 Regulation of p53 by TopBP1: a potential mechanism for p53 inactivation in cancer. Mol Cell Biol 29:2673–2693. doi:10.1128/MCB.01140-08. 19289498 PMC2682038

[35] Wright RH, Dornan ES, Donaldson MM, Morgan IM 2006 TopBP1 contains a transcriptional activation domain suppressed by two adjacent BRCT domains. Biochem J 400:573–582. doi:10.1042/BJ20060831. 16984230 PMC1698607

[36] Liu K, Luo Y, Lin FT, Lin WC 2004 TopBP1 recruits Brg1/Brm to repress E2F1-induced apoptosis, a novel pRb-independent and E2F1-specific control for cell survival. Genes Dev 18:673–686. doi:10.1101/gad.1180204. 15075294 PMC387242

[37] Liu K, Lin FT, Ruppert JM, Lin WC 2003 Regulation of E2F1 by BRCT domain-containing protein TopBP1. Mol Cell Biol 23:3287–3304. doi:10.1128/MCB.23.9.3287-3304.2003. 12697828 PMC153207

[38] Liu T, Lin YH, Leng W, Jung SY, Zhang H, Deng M, Evans D, Li Y, Luo K, Qin B, Qin J, Yuan J, Lou Z 2014 A divergent role of the SIRT1-TopBP1 axis in regulating metabolic checkpoint and DNA damage checkpoint. Mol Cell 56:681–695. doi:10.1016/j.molcel.2014.10.007. 25454945 PMC4386886

[39] Wang RH, Lahusen TJ, Chen Q, Xu X, Jenkins LM, Leo E, Fu H, Aladjem M, Pommier Y, Appella E, Deng CX 2014 SIRT1 deacetylates TopBP1 and modulates intra-S-phase checkpoint and DNA replication origin firing. Int J Biol Sci 10:1193–1202. doi:10.7150/ijbs.11066. 25516717 PMC4261203

[40] Fatoba ST, Tognetti S, Berto M, Leo E, Mulvey CM, Godovac-Zimmermann J, Pommier Y, Okorokov AL 2013 Human SIRT1 regulates DNA binding and stability of the Mcm10 DNA replication factor via deacetylation. Nucleic Acids Res 41:4065–4079. doi:10.1093/nar/gkt131. 23449222 PMC3627603

[41] Das D, Smith N, Wang X, Morgan IM 2017 The deacetylase SIRT1 regulates the replication properties of human papillomavirus 16 E1 and E2. J Virol 91:e00102-17. doi:10.1128/JVI.00102-17. 28275188 PMC5411580

[42] Utani K, Fu H, Jang SM, Marks AB, Smith OK, Zhang Y, Redon CE, Shimizu N, Aladjem MI 2017 Phosphorylated SIRT1 associates with replication origins to prevent excess replication initiation and preserve genomic stability. Nucleic Acids Res 45:7807–7824. doi:10.1093/nar/gkx468. 28549174 PMC5570034

[43] Anacker DC, Moody CA 2017 Modulation of the DNA damage response during the life cycle of human papillomaviruses. Virus Res 231:41–49. doi:10.1016/j.virusres.2016.11.006. 27836727 PMC5325762

[44] Anacker DC, Aloor HL, Shepard CN, Lenzi GM, Johnson BA, Kim B, Moody CA 2016 HPV31 utilizes the ATR-Chk1 pathway to maintain elevated RRM2 levels and a replication-competent environment in differentiating keratinocytes. Virology 499:383–396. doi:10.1016/j.virol.2016.09.028. 27764728 PMC5102796

[45] Gautam D, Moody CA 2016 Impact of the DNA damage response on human papillomavirus chromatin. PLoS Pathog 12:e1005613. doi:10.1371/journal.ppat.1005613. 27310012 PMC4910971

[46] Moody CA, Laimins LA 2009 Human papillomaviruses activate the ATM DNA damage pathway for viral genome amplification upon differentiation. PLoS Pathog 5:e1000605. doi:10.1371/journal.ppat.1000605. 19798429 PMC2745661

[47] Chappell WH, Gautam D, Ok ST, Johnson BA, Anacker DC, Moody CA 2016 Homologous recombination repair factors, Rad51 and BRCA1, are necessary for productive replication of human papillomavirus 31. J Virol 90:2639–2652. doi:10.1128/JVI.02495-15. PMC4810724 26699641

[48] Bristol ML, Wang X, Smith NW, Son MP, Evans MR, Morgan IM 2016 DNA damage reduces the quality, but not the quantity of human papillomavirus 16 E1 and E2 DNA replication. Viruses 8:175. doi:10.3390/v8060175. PMC4926195 27338449

[49] Anacker DC, Gautam D, Gillespie KA, Chappell WH, Moody CA 2014 Productive replication of human papillomavirus 31 requires DNA repair factor Nbs1. J Virol 88:8528–8544. doi:10.1128/JVI.00517-14. 24850735 PMC4135936

[50] Gillespie KA, Mehta KP, Laimins LA, Moody CA 2012 Human papillomaviruses recruit cellular DNA repair and homologous recombination factors to viral replication centers. J Virol 86:9520–9526. doi:10.1128/JVI.00247-12. 22740399 PMC3416172

[51] Sakakibara N, Chen D, McBride AA 2013 Papillomaviruses use recombination-dependent replication to vegetatively amplify their genomes in differentiated cells. PLoS Pathog 9:e1003321. doi:10.1371/journal.ppat.1003321. 23853576 PMC3701714

[52] Bristol ML, Das D, Morgan IM 2017 Why human papillomaviruses activate the DNA damage response (DDR) and how cellular and viral replication persists in the presence of DDR signaling. Viruses 9:268. doi:10.3390/v9100268. PMC5691620 28934154

[53] Yuan Z, Zhang X, Sengupta N, Lane WS, Seto E 2007 SIRT1 regulates the function of the Nijmegen breakage syndrome protein. Mol Cell 27:149–162. doi:10.1016/j.molcel.2007.05.029. 17612497 PMC2679807

[54] Duursma AM, Driscoll R, Elias JE, Cimprich KA 2013 A role for the MRN complex in ATR activation via TOPBP1 recruitment. Mol Cell 50:116–122. doi:10.1016/j.molcel.2013.03.006. 23582259 PMC3669687

[55] Palacios JA, Herranz D, De Bonis ML, Velasco S, Serrano M, Blasco MA 2010 SIRT1 contributes to telomere maintenance and augments global homologous recombination. J Cell Biol 191:1299–1313. doi:10.1083/jcb.201005160. 21187328 PMC3010065

[56] Langsfeld ES, Bodily JM, Laimins LA 2015 The deacetylase sirtuin 1 regulates human papillomavirus replication by modulating histone acetylation and recruitment of DNA damage factors NBS1 and Rad51 to viral genomes. PLoS Pathog 11:e1005181. doi:10.1371/journal.ppat.1005181. 26405826 PMC4583417

[57] Li K, Wang R, Lozada E, Fan W, Orren DK, Luo J 2010 Acetylation of WRN protein regulates its stability by inhibiting ubiquitination. PLoS One 5:e10341. doi:10.1371/journal.pone.0010341. 20428248 PMC2859066

[58] Li K, Casta A, Wang R, Lozada E, Fan W, Kane S, Ge Q, Gu W, Orren D, Luo J 2008 Regulation of WRN protein cellular localization and enzymatic activities by SIRT1-mediated deacetylation. J Biol Chem 283:7590–7598. doi:10.1074/jbc.M709707200. 18203716

[59] Uhl M, Csernok A, Aydin S, Kreienberg R, Wiesmuller L, Gatz SA 2010 Role of SIRT1 in homologous recombination. DNA Repair (Amst) 9:383–393. doi:10.1016/j.dnarep.2009.12.020. 20097625

[60] Vaitiekunaite R, Butkiewicz D, Krześniak M, Przybyłek M, Gryc A, Snietura M, Benedyk M, Harris CC, Rusin M 2007 Expression and localization of Werner syndrome protein is modulated by SIRT1 and PML. Mech Ageing Dev 128:650–661. doi:10.1016/j.mad.2007.09.004. 17996922

[61] Shamanna RA, Croteau DL, Lee JH, Bohr VA 2017 Recent advances in understanding Werner syndrome. F1000Res 6:1779. doi:10.12688/f1000research.12110.1. 29043077 PMC5621106

[62] Croteau DL, Popuri V, Opresko PL, Bohr VA 2014 Human RecQ helicases in DNA repair, recombination, and replication. Annu Rev Biochem 83:519–552. doi:10.1146/annurev-biochem-060713-035428. 24606147 PMC4586249

[63] Pichierri P, Ammazzalorso F, Bignami M, Franchitto A 2011 The Werner syndrome protein: linking the replication checkpoint response to genome stability. Aging (Albany, NY) 3:311–318. doi:10.18632/aging.100293. 21389352 PMC3091524

[64] Rossi ML, Ghosh AK, Bohr VA 2010 Roles of Werner syndrome protein in protection of genome integrity. DNA Repair (Amst) 9:331–344. doi:10.1016/j.dnarep.2009.12.011. 20075015 PMC2827637

[65] Edwards DN, Machwe A, Chen L, Bohr VA, Orren DK 2015 The DNA structure and sequence preferences of WRN underlie its function in telomeric recombination events. Nat Commun 6:8331. doi:10.1038/ncomms9331. 26420422 PMC4589872

[66] Taylor ER, Dornan ES, Boner W, Connolly JA, McNair S, Kannouche P, Lehmann AR, Morgan IM 2003 The fidelity of HPV16 E1/E2-mediated DNA replication. J Biol Chem 278:52223–52230. doi:10.1074/jbc.M308779200. 14559922

[67] Yuan Z, Seto E 2007 A functional link between SIRT1 deacetylase and NBS1 in DNA damage response. Cell Cycle 6:2869–2871. doi:10.4161/cc.6.23.5026. 18156798

[68] Lee SY, Lee H, Kim ES, Park S, Lee J, Ahn B 2015 WRN translocation from nucleolus to nucleoplasm is regulated by SIRT1 and required for DNA repair and the development of chemoresistance. Mutat Res 774:40–48. doi:10.1016/j.mrfmmm.2015.03.001. 25801465

[69] Taylor ER, Morgan IM 2003 A novel technique with enhanced detection and quantitation of HPV-16 E1- and E2-mediated DNA replication. Virology 315:103–109. doi:10.1016/S0042-6822(03)00588-9. 14592763

[70] Reinson T, Toots M, Kadaja M, Pipitch R, Allik M, Ustav E, Ustav M 2013 Engagement of the ATR-dependent DNA damage response at the human papillomavirus 18 replication centers during the initial amplification. J Virol 87:951–964. doi:10.1128/JVI.01943-12. 23135710 PMC3554080

[71] Kadaja M, Isok-Paas H, Laos T, Ustav E, Ustav M 2009 Mechanism of genomic instability in cells infected with the high-risk human papillomaviruses. PLoS Pathog 5:e1000397. doi:10.1371/journal.ppat.1000397. 19390600 PMC2666264

[72] Kadaja M, Sumerina A, Verst T, Ojarand M, Ustav E, Ustav M 2007 Genomic instability of the host cell induced by the human papillomavirus replication machinery. EMBO J 26:2180–2191. doi:10.1038/sj.emboj.7601665. 17396148 PMC1852791

[73] Fradet-Turcotte A, Bergeron-Labrecque F, Moody CA, Lehoux M, Laimins LA, Archambault J 2011 Nuclear accumulation of the papillomavirus E1 helicase blocks S-phase progression and triggers an ATM-dependent DNA damage response. J Virol 85:8996–9012. doi:10.1128/JVI.00542-11. 21734051 PMC3165840

[74] Sakakibara N, Mitra R, McBride AA 2011 The papillomavirus E1 helicase activates a cellular DNA damage response in viral replication foci. J Virol 85:8981–8995. doi:10.1128/JVI.00541-11. 21734054 PMC3165833

[75] King LE, Dornan ES, Donaldson MM, Morgan IM 2011 Human papillomavirus 16 E2 stability and transcriptional activation is enhanced by E1 via a direct protein-protein interaction. Virology 414:26–33. doi:10.1016/j.virol.2011.03.002. 21458836

[76] Su F, Bhattacharya S, Abdisalaam S, Mukherjee S, Yajima H, Yang Y, Mishra R, Srinivasan K, Ghose S, Chen DJ, Yannone SM, Asaithamby A 2016 Replication stress induced site-specific phosphorylation targets WRN to the ubiquitin-proteasome pathway. Oncotarget 7:46–65. doi:10.18632/oncotarget.6659. 26695548 PMC4807982

[77] Taylor ER, Boner W, Dornan ES, Corr EM, Morgan IM 2003 UVB irradiation reduces the half-life and transactivation potential of the human papillomavirus 16 E2 protein. Oncogene 22:4469–4477. doi:10.1038/sj.onc.1206746. 12881703

[78] Evans MR, James CD, Loughran O, Nulton TJ, Wang X, Bristol ML, Windle B, Morgan IM 2017 An oral keratinocyte life cycle model identifies novel host genome regulation by human papillomavirus 16 relevant to HPV positive head and neck cancer. Oncotarget 8:18892–81909. doi:10.18632/oncotarget.18328. PMC5669857 29137231

[79] Piboonniyom SO, Duensing S, Swilling NW, Hasskarl J, Hinds PW, Munger K 2003 Abrogation of the retinoblastoma tumor suppressor checkpoint during keratinocyte immortalization is not sufficient for induction of centrosome-mediated genomic instability. Cancer Res 63:476–483. 12543805

[80] King LE, Fisk JC, Dornan ES, Donaldson MM, Melendy T, Morgan IM 2010 Human papillomavirus E1 and E2 mediated DNA replication is not arrested by DNA damage signalling. Virology 406:95–102. doi:10.1016/j.virol.2010.06.033. 20673941

[81] Yang H, Bi Y, Xue L, Wang J, Lu Y, Zhang Z, Chen X, Chu Y, Yang R, Wang R, Liu G 2015 Multifaceted modulation of SIRT1 in cancer and inflammation. Crit Rev Oncog 20:49–64. doi:10.1615/CritRevOncog.2014012374. 25746104

[82] Gorospe M, de Cabo R 2008 AsSIRTing the DNA damage response. Trends Cell Biol 18:77–83. doi:10.1016/j.tcb.2007.11.007. 18215521 PMC8483772

[83] Oberdoerffer P, Michan S, McVay M, Mostoslavsky R, Vann J, Park SK, Hartlerode A, Stegmuller J, Hafner A, Loerch P, Wright SM, Mills KD, Bonni A, Yankner BA, Scully R, Prolla TA, Alt FW, Sinclair DA 2008 SIRT1 redistribution on chromatin promotes genomic stability but alters gene expression during aging. Cell 135:907–918. doi:10.1016/j.cell.2008.10.025. 19041753 PMC2853975

[84] Vaziri H, Dessain SK, Ng Eaton E, Imai SI, Frye RA, Pandita TK, Guarente L, Weinberg RA 2001 hSIR2(SIRT1) functions as an NAD-dependent p53 deacetylase. Cell 107:149–159. doi:10.1016/S0092-8674(01)00527-X. 11672523

[85] Palermo V, Rinalducci S, Sanchez M, Grillini F, Franchitto A, Pichierri P 2017 Way out/way in: how the relationship between WRN and CDK1 may change the fate of collapsed replication forks. Mol Cell Oncol 4:e1268243. doi:10.1080/23723556.2016.1268243. 28197541 PMC5286962

[86] Palermo V, Rinalducci S, Sanchez M, Grillini F, Sommers JA, Brosh RM Jr, Zolla L, Franchitto A, Pichierri P 2016 CDK1 phosphorylates WRN at collapsed replication forks. Nat Commun 7:12880. doi:10.1038/ncomms12880. 27634057 PMC5028418

[87] Iannascoli C, Palermo V, Murfuni I, Franchitto A, Pichierri P 2015 The WRN exonuclease domain protects nascent strands from pathological MRE11/EXO1-dependent degradation. Nucleic Acids Res 43:9788–9803. doi:10.1093/nar/gkv836. 26275776 PMC4787784

[88] Basile G, Leuzzi G, Pichierri P, Franchitto A 2014 Checkpoint-dependent and independent roles of the Werner syndrome protein in preserving genome integrity in response to mild replication stress. Nucleic Acids Res 42:12628–12639. doi:10.1093/nar/gku1022. 25352544 PMC4227752

[89] Murfuni I, Nicolai S, Baldari S, Crescenzi M, Bignami M, Franchitto A, Pichierri P 2013 The WRN and MUS81 proteins limit cell death and genome instability following oncogene activation. Oncogene 32:610–620. doi:10.1038/onc.2012.80. 22410776

[90] Pichierri P, Nicolai S, Cignolo L, Bignami M, Franchitto A 2012 The RAD9-RAD1-HUS1 (9.1.1) complex interacts with WRN and is crucial to regulate its response to replication fork stalling. Oncogene 31:2809–2823. doi:10.1038/onc.2011.468. 22002307 PMC3272477

[91] Ammazzalorso F, Pirzio LM, Bignami M, Franchitto A, Pichierri P 2010 ATR and ATM differently regulate WRN to prevent DSBs at stalled replication forks and promote replication fork recovery. EMBO J 29:3156–3169. doi:10.1038/emboj.2010.205. 20802463 PMC2944071

[92] Hong S, Cheng S, Iovane A, Laimins LA 2015 STAT-5 regulates transcription of the topoisomerase IIβ-binding protein 1 (TopBP1) gene to activate the ATR pathway and promote human papillomavirus replication. mBio 6:e02006. doi:10.1128/mBio.02006-15. 26695634 PMC4701836

[93] Morgan IM, DiNardo LJ, Windle B 2017 Integration of human papillomavirus genomes in head and neck cancer: is it time to consider a paradigm shift? Viruses 9:208. doi:10.3390/v9080208. PMC5580465 28771189

[94] Nulton TJ, Olex AL, Dozmorov M, Morgan IM, Windle B 2017 Analysis of the cancer genome atlas sequencing data reveals novel properties of the human papillomavirus 16 genome in head and neck squamous cell carcinoma. Oncotarget 8:17684–17699. doi:10.18632/oncotarget.15179. 28187443 PMC5392278

[95] Anayannis NV, Schlecht NF, Ben-Dayan M, Smith RV, Belbin TJ, Ow TJ, Blakaj DM, Burk RD, Leonard SM, Woodman CB, Parish JL, Prystowsky MB 2018 Association of an intact E2 gene with higher HPV viral load, higher viral oncogene expression, and improved clinical outcome in HPV16 positive head and neck squamous cell carcinoma. PLoS One 13:e0191581. doi:10.1371/journal.pone.0191581. 29451891 PMC5815588

[96] Ramqvist T, Mints M, Tertipis N, Nasman A, Romanitan M, Dalianis T 2015 Studies on human papillomavirus (HPV) 16 E2, E5 and E7 mRNA in HPV-positive tonsillar and base of tongue cancer in relation to clinical outcome and immunological parameters. Oral Oncol 51:1126–1131. doi:10.1016/j.oraloncology.2015.09.007. 26421862

[97] Bouvard V, Storey A, Pim D, Banks L 1994 Characterization of the human papillomavirus E2 protein: evidence of trans-activation and trans-repression in cervical keratinocytes. EMBO J 13:5451–5459. doi:10.1002/j.1460-2075.1994.tb06880.x. 7957111 PMC395503

[98] Lee JH, Yi SM, Anderson ME, Berger KL, Welsh MJ, Klingelhutz AJ, Ozbun MA 2004 Propagation of infectious human papillomavirus type 16 by using an adenovirus and Cre/LoxP mechanism. Proc Natl Acad Sci U S A 101:2094–2099. doi:10.1073/pnas.0308615100. 14769917 PMC357057

