# Correction for Morgan et al., “Werner Helicase Control of Human Papillomavirus 16 E1-E2 DNA Replication Is Regulated by SIRT1 Deacetylation”

**DOI:** 10.1128/mBio.01635-19

**Published:** 2019-08-13

**Authors:** Dipon Das, Molly L. Bristol, Nathan W. Smith, Claire D. James, Xu Wang, Pietro Pichierri, Iain M. Morgan

**Affiliations:** a Department of Oral and Craniofacial Molecular Biology, VCU Philips Institute for Oral Health Research, Virginia Commonwealth University School of Dentistry, Richmond, Virginia, USA; b Department of Environment and Health, Istituto Superiore di Sanità, Rome, Italy; c VCU Massey Cancer Center, Richmond, Virginia, USA

## ERRATUM

Volume 10, no. 2, e00263-19, 2019, https://doi.org/10.1128/mBio.00263-19. It has come to our attention that in this study, the isogenic TERT immortalized keratinocyte lines that were used in Fig. 6 are not NOK but are in fact N/Tert-1. We apologize for this honest error that has been corrected in the revised Fig. 6. None of the conclusions from this work are compromised in any way.

**FIG 6:**
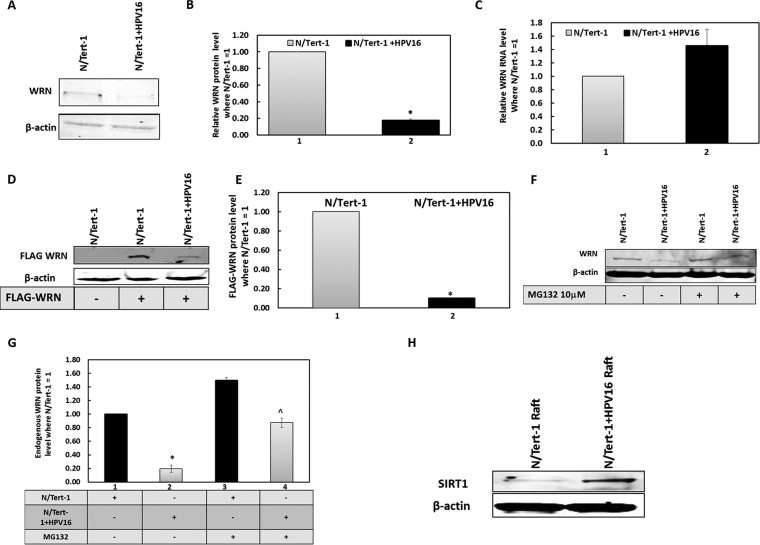
WRN protein turnover is regulated by the entire HPV16 genome in nontransformed keratinocytes, similarly to E1-E2 replication in C33a cells. (A) N/Tert-1 cells and N/Tert-1 plus HPV16 (cells that contain episomal HPV16 genomes and support late stages of the viral life cycle [78]) were blotted for endogenous WRN protein levels. (B) Duplicate experiments of that shown in panel A were quantitated, and there is a significant decrease in WRN protein levels in the presence of HPV16 (*, *P* value was less than 0.05; standard error bars are shown). (C) This reduction is not due to a reduction in WRN RNA levels. Results from an average of three independent experiments are shown from reverse transcriptase quantitative PCR, and there is no significant difference in WRN RNA in the absence or presence of HPV16 (standard error bars are shown). (D) N/Tert-1 and N/Tert-1 plus HPV16 were transiently transfected with the FLAG-WRN expression vector, and 48 h later, protein extracts were prepared and FLAG Western blotting was carried out. (E) Duplicate experiments of that shown in panel D were quantitated, and there is a significant decrease in FLAG-WRN levels in the presence of HPV16 (*, *P* value was less than 0.05; standard error bars are shown). (F) N/Tert-1 and N/Tert 1 plus HPV16 were treated with MG132 for 18 h prior to preparation of protein extracts and Western blotting for endogenous WRN. (G) The results of duplicate experiments of that shown in panel F were quantitated, and there is a significant increase in WRN protein levels in N/Tert-1 plus HPV16 but not N/Tert-1 following MG132 treatment (*, *P* value was less than 0.05; standard error bars are shown). (H) Protein extracts were prepared from N/Tert-1 and N/Tert-1 plus HPV16 cells that had been subjected to organotypic rafting. HPV16 induces levels of SIRT1 in the N/Tert-1 cells, as evidenced by the large increase in SIRT1 protein detection.

