# Supplementary figures and images for: Werner Helicase Control of Human Papillomavirus 16 E1-E2 DNA Replication Is Regulated by SIRT1 Deacetylation
Source: mBio. 2019 Mar 19;10(2):e00263-19. doi: 10.1128/mBio.00263-19 (PMC6426601; doi:10.1128/mBio.00263-19)

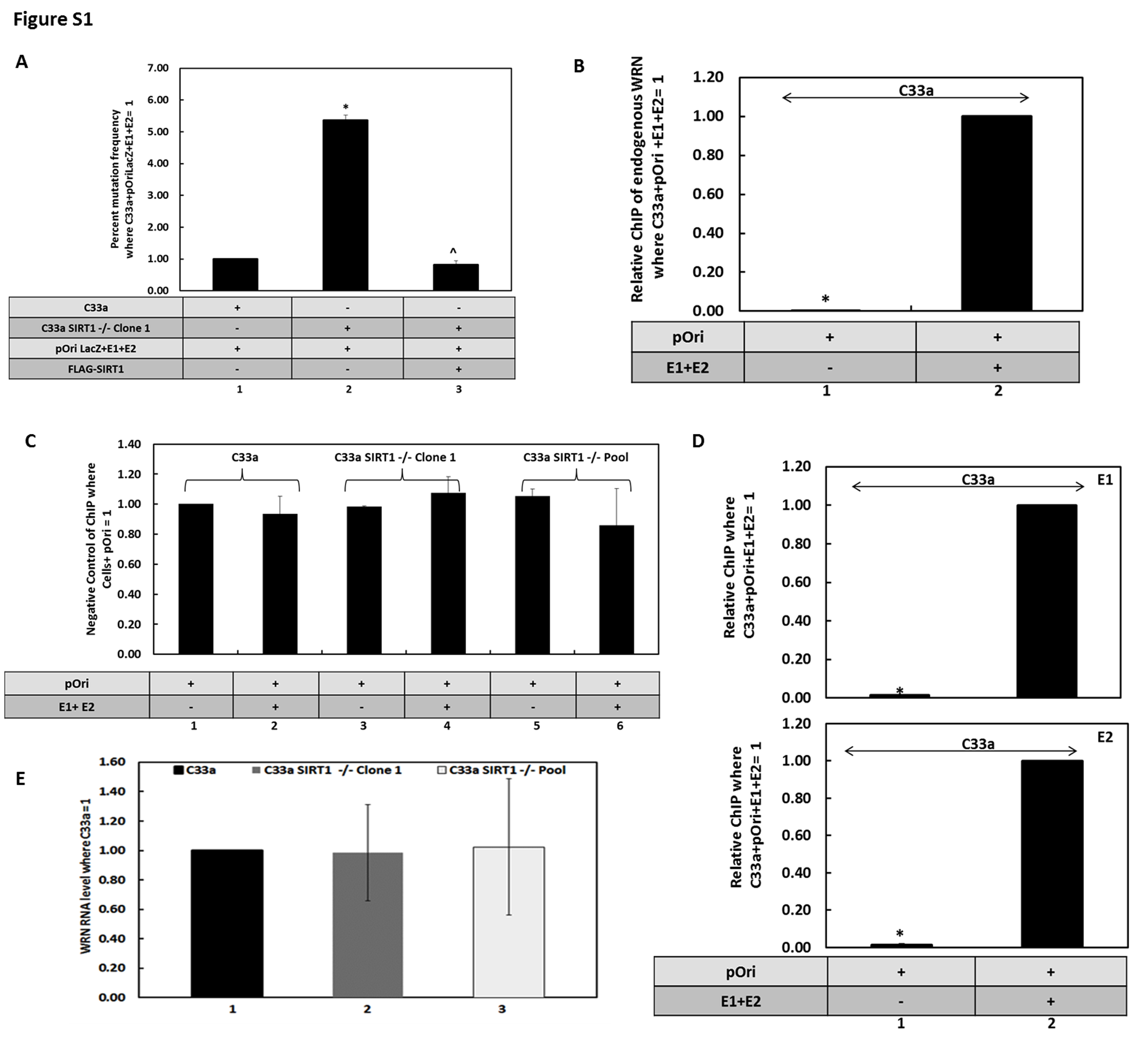

Supplement: FIG S1 [file mBio.00263-19-sf001.tif]

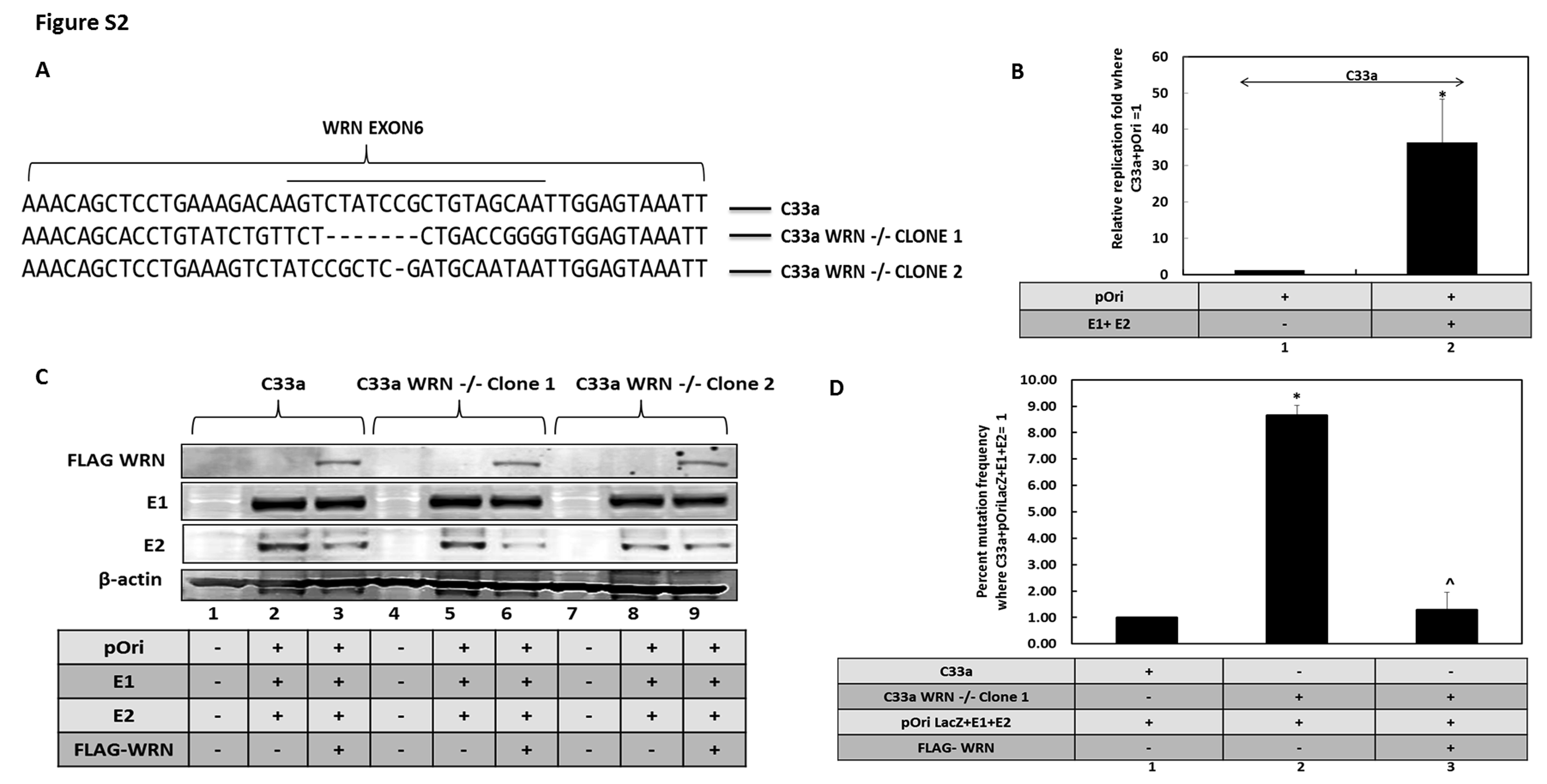

Supplement: FIG S2 [file mBio.00263-19-sf002.tif]

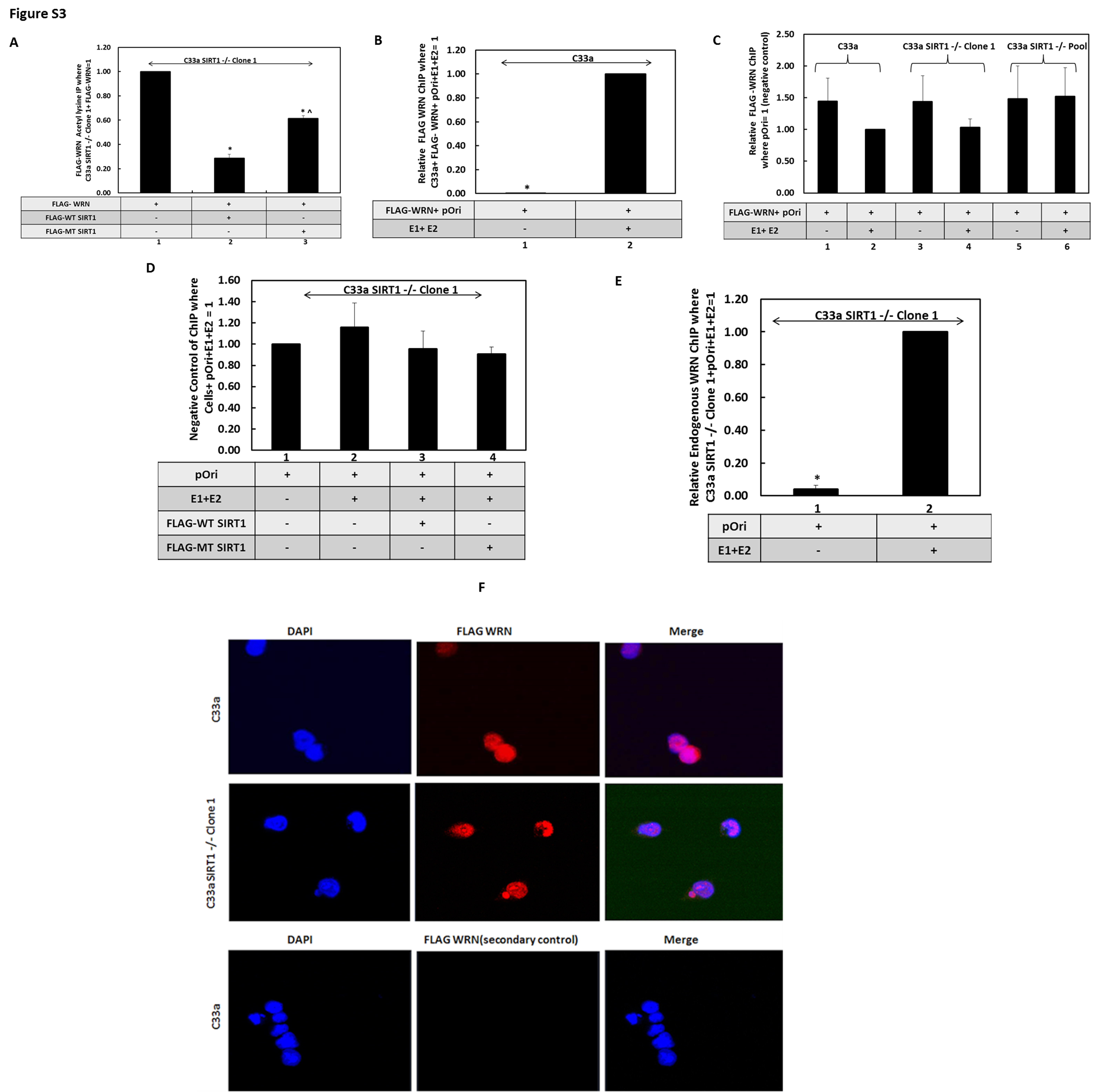

Supplement: FIG S3 [file mBio.00263-19-sf003.tif]

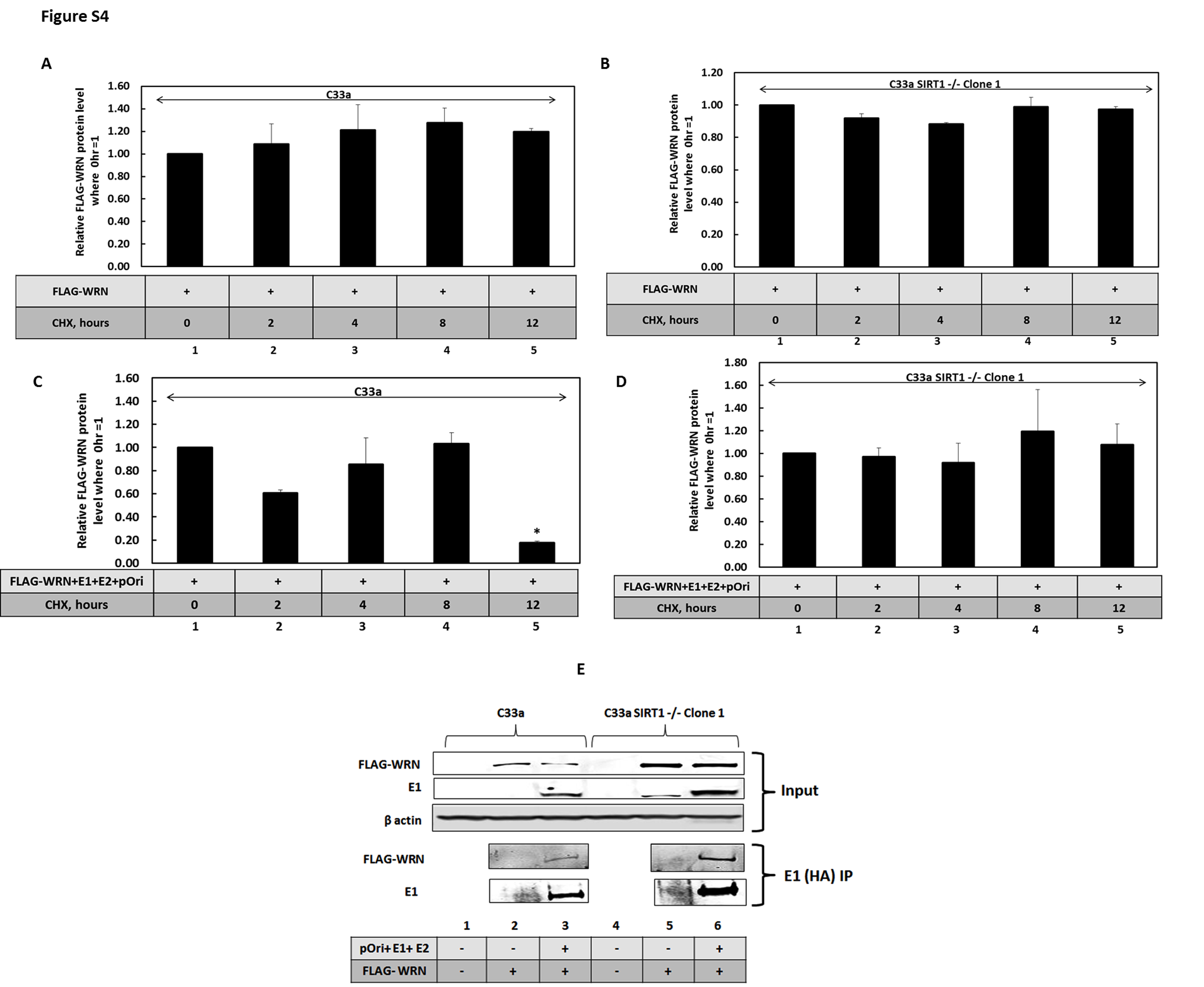

Supplement: FIG S4 [file mBio.00263-19-sf004.tif]
